# Collagen type IV alpha 6 promotes tumor progression and chemoresistance in ovarian cancer by activating the discoidin domain receptor 1 pathway

**DOI:** 10.1038/s41389-025-00565-2

**Published:** 2025-07-02

**Authors:** Yi-Hui Wu, Pei-Ying Wu, Yu-Fang Huang, Chien-Chin Chen, Soon-Cen Huang, Chou Cheng-Yang

**Affiliations:** 1 Department of Medical Research, Chi Mei Medical Center, Liouying, Tainan, Taiwan; 2 Center of General Education, Min-Hwei Junior College of Health Care Management, Tainan, Taiwan; 3 Department of Obstetrics and Gynecology, National Cheng Kung University Hospital, College of Medicine, National Cheng Kung University, Tainan, Taiwan; 4 Department of Pathology, Ditmanson Medical Foundation Chia-Yi Christian Hospital, Chia-Yi, Taiwan; 5 Department of Cosmetic Science, Chia Nan University of Pharmacy and Science, Tainan, Taiwan; 6 Doctoral Program in Translational Medicine, National Chung Hsing University, Taichung, Taiwan; 7 Department of Biotechnology and Bioindustry Sciences, College of Biotechnology, National Cheng Kung University, Tainan, Taiwan; 8 Department of Obstetrics and Gynecology, Chi Mei Medical Center, Liouying, Tainan Taiwan

**Keywords:** Ovarian cancer, Prognostic markers

## Abstract

Biomarkers that predict disease progression may assist in the development of better therapeutic strategies for aggressive cancers, such as ovarian cancer. This study aimed to investigate the role of collagen type IV alpha 6 (COL4A6) in cell invasiveness, tumor formation, chemoresistance, and the prognostic impact of COL4A6 expression in ovarian cancer. COL4A6 regulated discoidin domain receptor 1 (DDR1)/p-DDR1 expression through the binding of E2F transcription factor 1 (E2F) to its putative DDR1 promoter binding site, suggesting that the E2F–DDR1 axis is upregulated by COL4A6. Pharmacological inhibition of DDR1 abrogated COL4A6-dependent cell invasiveness and chemoresistance. COL4A6 regulated cell invasion via the E2F1/DDR1/focal adhesion kinase axis; in contrast, COL4A6 regulated cell sensitivity to cisplatin via the DDR1/nuclear factor-kappa B axis. DDR1-IN-1 increased cell sensitivity to cisplatin, synergized with cisplatin to suppress the invasive ability and oncogenic potential of ovarian cancer cells, and decreased tumor formation in mouse xenografts. High COL4A6 mRNA levels were associated with advanced disease stages and poor chemotherapy response. The 5-year recurrence-free and overall survival rates were significantly lower in patients with high tissue COL4A6 mRNA expression levels than in those with low expression levels. COL4A6 may promote tumor aggressiveness and chemoresistance via the E2F/DDR1 axis, and COL4A6 expression can predict clinical outcomes in patients with ovarian cancer. DDR1 should be targeted in patients with COL4A6-positive tumors.

## Introduction

Epithelial ovarian carcinoma (EOC) is the most fatal gynecological malignancy [1]. Most patients with EOC are diagnosed at an advanced stage, and although most initially respond to cytoreductive surgery and platinum-based chemotherapy, many patients eventually relapse, develop chemoresistant tumors, and die from the disease [2, 3]. Because the empirical incorporation of additional cytotoxic agents against ovarian cancer (OC) does not improve prognosis [4], an understanding of the key signaling pathways responsible for chemoresistance and disease progression is required to develop new treatment strategies.

Collagen type IV alpha 6 (COL4A6), a member of the collagen family, is a component of the extracellular matrix (ECM). The ECM, a crucial element of the basement membrane, plays a pivotal role in shaping the tumor microenvironment [5]. COL4A6 deletion is associated with the molecular pathogenesis of uterine leiomyomas [6], diffuse esophageal leiomyomatosis [7], and Alport syndrome [8]. COL4A6 downregulation correlates with metastasis in various cancers, including melanoma, colorectal cancer, follicular thyroid cancer, prostate cancer, basal cell carcinoma, and breast cancer [9–14]. Conversely, COL4A6 acts as an oncogene in gastric cancer [15], suggesting that its function varies with the tissue type. However, COL4A6 role in OC requires further investigation. Thus, this study investigated COL4A6’s role in the invasive activity of OC cells, cell sensitivity to cisplatin, and the clinical outcomes of patients with OC. It provides direct functional evidence that COL4A6 promotes invasiveness, chemoresistance, and disease progression in OC. Furthermore, the molecular mechanisms underlying COL4A6-enhanced cancer cell invasion and chemoresistance were elucidated to provide an understanding of its mode of action.

## Results

### Clinical correlations and prognostic significance of COL4A6 mRNA expression in patients with ovarian cancer

**Fig. 1:**
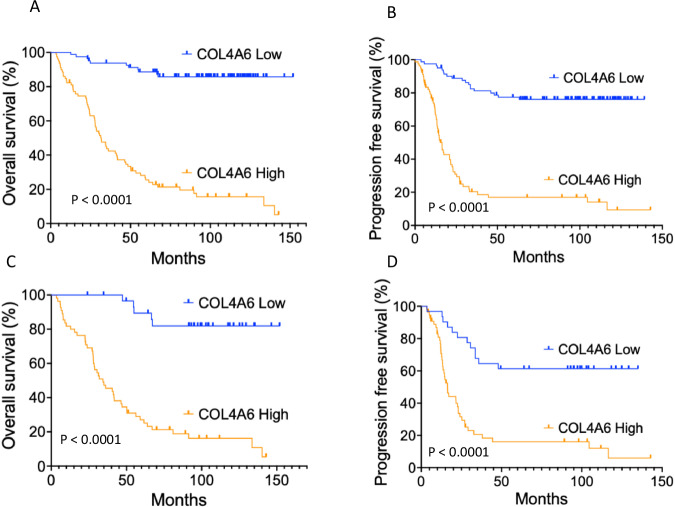
Kaplan–Meier curves stratified based on the COL4A6 mRNA level and analyzed using a log-rank test. **A** OS in all 160 patients with OC, (**B**) PFS in all 160 patients with OC, (**C**) OS in 86 patients with high-grade serous subtype, **D** PFS in 86 patients with high-grade serous subtype.

*COL4A6* mRNA expression and the characteristics of 160 EOC cases are shown in Table 1. The median follow-up period was 66.1 (range, 3.5–151.9) months. During follow-up, 80 (50.0%) patients experienced cancer progression, and 77 (48.1%) patients died. *COL4A6* mRNA expression levels in tumors were evaluated using real-time RT-PCR. A cut-off value of 1833 was used to categorize the tumors into groups with high or low *COL4A6* mRNA levels. The cut-off value of 1833 for COL4A6 mRNA expression was determined using a Receiver Operating Characteristic (ROC) curve analysis, with progression-free survival (PFS) status as the clinical outcome. Specifically, the ROC curve was used to identify the optimal threshold that best distinguished patients with longer vs. shorter PFS. The cut-off point of 1833 was selected based on Youden’s index, achieving a sensitivity of 0.70 and a specificity of 0.79. This suggests a good balance between true positive and true negative rates for predicting PFS outcomes. This approach will provide a data-driven and clinically meaningful way to stratify COL4A6 expression into high and low categories. High *COL4A6* mRNA levels were significantly correlated with advanced stage and serous type when compared with the low *COL4A6* mRNA levels (advanced stage 79.78% vs. early stage 20.22%, *P* = 0.0001; serous type 63.95% vs. non-serous 36.05%, *P* = 0.0001). Additionally, high *COL4A6* expression levels were significantly associated with a poor response to chemotherapy and a higher resistance rate to platinum chemotherapy (complete/partial response rate in high *COL4A6* level vs. low *COL4A6* level, 52 [45.61%] vs. 62 [54.39%], *P* = 0.0001; platinum-free interval ≤6 months vs. >6 months, *P* = 0.0001) (Table 1). In all 160 patients with OC and 86 patients with high-grade serous subtype, patients with high *COL4A6* mRNA levels had significantly shorter Overall Survival (OS) and PFS compared with those with low *COL4A6* mRNA levels (Figure 1).

**Table 1:** Clinico-pathological characteristics and their associations with COL4A6 mRNA levels in ovarian cancer patients.

Variable	Patient No. (%)	COL4A6 mRNA		*P*-value
		Low No. (%)	High No. (%)	
Number	160	81(50.6)	79(49.4)	
**Age (**mean ± SD)	54.13 ± 12.21	51.51 ± 13.29	56.82 ± 10.40	0.0056
**Stage**				0.0001
Early (I + II)	71	63(88.73)	8(11.27)	
Advanced (III + IV)	89	18(20.22)	71(79.78)	
**Histology**				0.0001
Serous	86	31 (36.05)	55 (63.95)	
Non-serous	74	50 (61.73)	24 (30.38)	
**Chemotherapy Response**				0.0001
CR & PR	114	62 (54.39)	52 (45.61)	
SD & PD	25	3 (12)	22 (88)	
**Progression-free interval**				0.0001
>6 months	131	78 (59.5)	53 (40.5)	
≤6 months	28	2 (7.1)	26 (92.9)	
**Tumor recurrence**				0.0001
No	80	62 (77.5)	18 (22.5)	
Yes	80	19 (23.75)	61 (76.25)	
**Death**				0.0001
No	83	70 (84.3)	13 (15.7)	
Yes	77	11 (14.3)	66 (85.7)	

Data was presented as frequency (percentage).

Categorical data was analyzed by Chi-square test or Fisher’s exact test. Continous categrory was analyzed by *t*-test.

COL4A6 level <1833 (low) and ≥1833 (high).

*CR* complete response, *PR* partial response, *SD* stable disease, *PD* progressive disease.

### COL4A6 regulates ovarian cancer aggressiveness

**Fig. 2:**
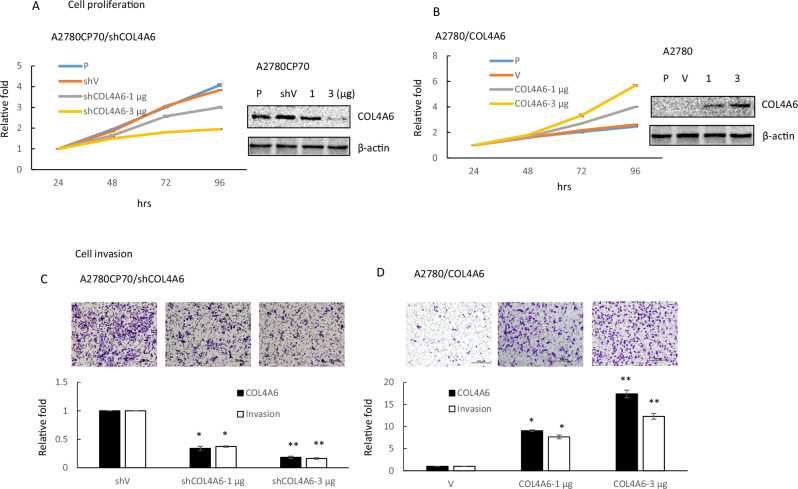
COL4A6 regulates ovarian cancer cell proliferation and invasion. **A** A2780CP70 cells were transfected with different quantities of a COL4A6 knockdown plasmid. After 48 h, COL4A6 expression was evaluated by western blotting in whole lysates. β-actin was used as a protein loading control. The cell doubling time was measured using the MTT assay. **B** A2780 cells were transfected with different quantities of a COL4A6-expression plasmid. After 48 h, COL4A6 expression was evaluated by western blotting in whole lysates. β-actin was used as a protein loading control. The cell doubling time was measured using the MTT assay. **C** A2780CP70 cells were transfected with different quantities of a COL4A6 knockdown plasmid. The cells were trypsinized and collected from the dishes. Samples consisting of 1 × 10^4^ cells were seeded into Transwells to evaluate invasion capacity. COL4A6 mRNA expression was evaluated by qRT-PCR. All data represent the means ± standard deviations of three separate experiments. **D** A2780 cells were transfected with different quantities of a COL4A6-expression plasmid. The cells were trypsinized and collected from the dishes. Samples consisting of 1 × 10^4^ cells were seeded into Transwells to evaluate invasion capacity. COL4A6 mRNA expression was evaluated by qRT-PCR. All data represent the means ± standard deviations of three separate experiments. *P*-values were determined using the Student’s *t*-test. ^*^P < 0.05 or ^**^P < 0.005^,^ relative to control cells treated with shControl or V.

To examine whether COL4A6 modulates the growth and invasive ability of OC cells, A2780CP70 cells were transfected with a siRNA specific for the *COL4A6* gene (shCOL4A6), and a *COL4A6* cDNA plasmid was introduced into COL4A6-low expressing A2780 cells to induce its overexpression. The data showed that COL4A6 protein levels were reduced in cells upon knockdown at different concentrations (Fig. 2A, right panel). COL4A6-knockdown A2780CP70 cell viability was markedly reduced (Fig. 2A, left panel, 41.01 ± 0.36 h for shCOL4A6-3 μg vs. 27.10 ± 0.80 h for shV, *P* < 0.005). Conversely, COL4A6 expression was increased in COL4A6-overexpressing A2780 cells (Fig. 2B, right panel). COL4A6-overexpressing A2780 cell viability increased (Fig. 2B, left panel, 36 ± 0.85 h for COL4A6-3 μg vs. 28.53 ± 1.13 h for V, *P* < 0.05). Real-time RT-PCR assays further confirmed that COL4A6 mRNA expression was reduced in COL4A6-knockdown cells (Fig. 2C, lower panel) and increased in COL4A6-overexpressing cells (Fig. 2D, lower panel). Transwell invasion assays indicated that COL4A6-knockdown cell invasive ability was impaired (Fig. 2C) and was increased in COL4A6-overexpressing cells (Fig. 2D). COL4A6 expression is associated with the cell invasion ability and growth of OC cells.

### COL4A6 regulates cell invasion ability via the E2F1/DDR1/FAK axis

**Fig. 3:**
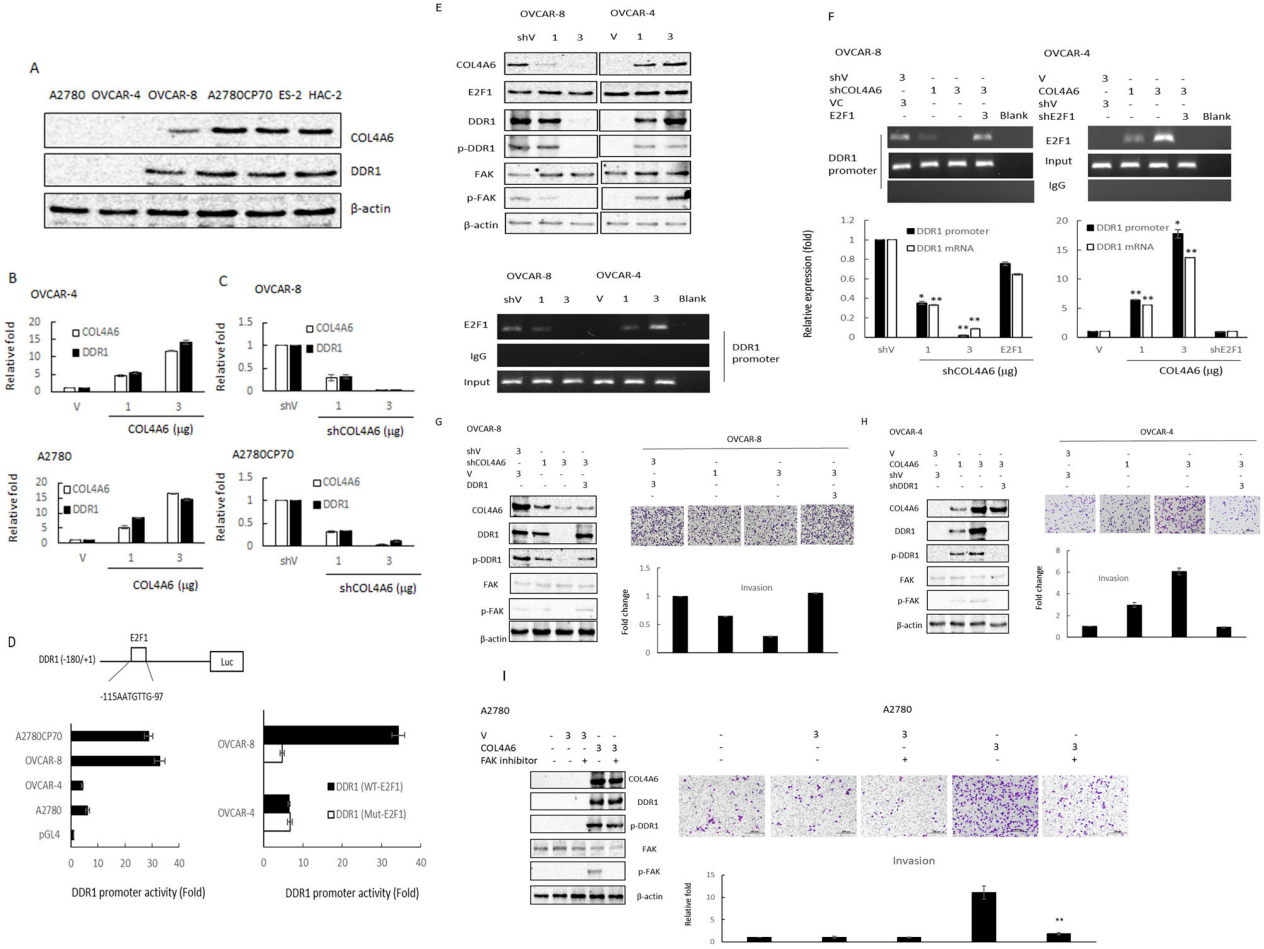
COL4A6 regulates DDR1 transcription by the upregulation of E2F1 binding to the DDR1 promoter, and COL4A6 regulates cell invasion ability via the E2F1/DDR1/FAK axis. **A** The protein expression levels of COL4A6 and DDR1 in a panel of ovarian cancer cells were evaluated by western blotting. β-actin served as a protein loading control. All experiments were performed in triplicate. **B** The mRNA expression levels of *COL4A6* and *DDR1* in OVCAR-4 and A2780 cells transfected with different quantities of a COL4A6 plasmid (1 and 3 µg) were evaluated by qRT-PCR. All experiments were performed in triplicate. **C** The mRNA-expression levels of COL4A6 and DDR1 in OVCAR-8 and A2780CP70 cells transfected with different quantities of a COL4A6 knockdown plasmid (1 and 3 µg) were evaluated by qRT-PCR. All experiments were performed in triplicate. **D** Schematic diagram of the DDR1 promoter-driven luciferase reporter: DDR1 (–180/ + 1). E2F1 has a putative E2F1-binding site. The promoter constructs were co-transfected into A2780, OVCAR-8, OVCAR-4, and A2780 cells, and luciferase reporter assays were performed 48 h later. The one putative E2F1-binding site on DDR1 (–180/ + 1) was changed by site-directed mutagenesis, and the mutated promoters were transfected into OVCAR-8 and OVCAR-4 cells to evaluate reporter activity. (E) Upper panel: Western blotting was performed to evaluate COL4A6, E2F1, DDR1, p-DDR1, FAK, and p-FAK protein expression in COL4A6 knockdown OVCAR-8 and COL4A6-overexpressing OVCAR-4 cells. Lower panel: The binding of E2F1 to the DDR1 promoter was evaluated by ChIP in COL4A6 knockdown OVCAR-8 and COL4A6-overexpressing OVCAR-4 cells. Chromatin was isolated and immunoprecipitated using an anti-E2F1 antibody. **F** Upper panel: The binding of E2F1 to the DDR1 promoter was evaluated by ChIP in OVCAR-8 cells transiently transfected with different quantities of a COL4A6 knockdown plasmid (1 and 3 µg) and an E2F1 cDNA plasmid (3 µg), and OVCAR-4 cells were transfected with different quantities of a COL4A6 plasmid (1 and 3 µg) and an E2F1 knockdown plasmid (3 µg). Chromatin was isolated and immunoprecipitated using an anti-E2F1 antibody. Lower panel: OVCAR-8 cells were transfected with a COL4A6 knockdown plasmid and an E2F1 cDNA plasmid, and OVCAR-4 cells were transfected with a COL4A6 cDNA plasmid and E2F1 knockdown plasmid. DDR1 mRNA levels and promoter activities were evaluated by performing qRT-PCR and luciferase assays, respectively. All experiments were performed in triplicate. *P* values were determined using the Student’s *t*-test. ^*^P < 0.05 or ^**^P < 0.005^,^ relative to the control cells treated with shV or VC. **G** Left panel: OVCAR-8 cells were transiently transfected with COL4A6 knockdown plasmid and a DDR1 cDNA plasmid. After 48 h, COL4A6, DDR1, p-DDR1, FAK, and p-FAK expressions were evaluated by western blotting in whole lysates after both treatment conditions. β-actin was used as a protein loading control. Right panel: The cells were trypsinized and collected from the dishes. Samples consisting of 1 × 10^4^ cells were seeded into Transwells to evaluate invasion capacity. All data represent the means ± standard deviations of three separate experiments. (H) Left panel: OVCAR-4 cells were transiently transfected with COL4A6-expression plasmid and a DDR1 knockdown plasmid. After 48 h, COL4A6, DDR1, p-DDR1, FAK, and p-FAK expression was evaluated by western blotting in whole lysates after both treatment conditions. β-actin was used as a protein loading control. Right panel: The cells were trypsinized and collected from the dishes. Samples consisting of 1 × 10^4^ cells were seeded into Transwells to evaluate invasion capacity. All data represent the means ± the standard deviations of three separate experiments. **I** Left panel: western blotting was performed to evaluate COL4A6, DDR1, p-DDR1, FAK, and p-FAK protein expression after treatment of A2780-COL4A6 expression cells for 24 h with FAK inhibitor. β–actin was the loading control. Right panel: In vitro invasion activity of A2780-COL4A6 expression cells after treatments. All data represent the mean ± the standard deviation of three separate experiments.

Collagen binding to DDR1 stimulates DDR1 phosphorylation, activating kinase activity that can initiate signaling [16]. DDR1 facilitates migration and invasion in breast cancer cells via FAK signaling activation [17]. FAK is one major effector that transduces signals from COL4A6 [18]. Thus, we hypothesized that COL4A6-mediated enhancement of cell aggressiveness might be regulated via DDR1/FAK activation. First, the relationship between COL4A6 and DDR1 expression in a panel of OC cells was examined. OC is a highly heterogeneous disease with different histological subtypes and molecular compositions. Thus, a panel of six OC cell lines was tested, including A2780, A2780CP70 (endometrioid histology), OVCAR-4, OVCAR-8 (serous histology), ES-2 and HAC2 (clear cell histology). Western blotting showed that COL4A6 expression positively correlated with DDR1 expression (Fig. 3A). The expressions of DDR1 and COL4A6 were found to be higher in the ES-2, HAC-2, and cisplatin-resistant cell line A2780CP70 (Fig. 3A).

To examine whether DDR1 expression is regulated by COL4A6 in OC cells, OVCAR-4 and A2780 cells were transfected with a COL4A6 cDNA plasmid, whereas OVCAR-8 and A2780CP70 cells were transfected with shRNA targeting COL4A6. As expected, DDR1 mRNA expression was elevated in COL4A6-overexpressing cells (Fig. 3B) and decreased in COL4A6-knockdown cells (Fig. 3C). These results indicate that COL4A6 regulates DDR1 expression in OC cells.

However, a direct association between COL4A6 and DDR1 has not yet been demonstrated. To further explore the mechanism by which COL4A6 increases DDR1 transcription, a fragment spanning positions from –180 to +1 relative to the DDR1 transcription start site was amplified using PCR, sequenced, and cloned into a luciferase reporter plasmid. As shown in Fig. 3D, the reporter activity of the DDR1 promoter upon transfection of the –180 to +1 fragment in A2780CP70 and OVCAR-8 cells was significantly higher than that in OVCAR-4 and A2780 cells transfected with the same fragment (Fig. 3D, left panel). Data from a previous study indicated the presence of an E2F1-binding site in the DDR1 promoter that regulates DDR1 transcription [19]. Whether DDR1 expression activation by COL4A6 was mediated by its increased E2F1-binding activity was investigated. Therefore, the E2F1-binding site in the DDR1 promoter was mutated using site-directed mutagenesis. The DDR1 (–180/ + 1) promoter with a mutated E2F1 binding site significantly lowered DDR1 promoter activity compared with the wild-type promoter in OVCAR-8 cells (Fig. 3D, right panel).

The results further showed increased total DDR1, DDR1, and FAK phosphorylations in COL4A6-overexpressing OVCAR-4 cells and decreased total DDR1, DDR1, and FAK phosphorylations in COL4A6-knockdown OVCAR-8 cells (Fig. 3E, upper panel). ChIP analysis further indicated that E2F1 binding to the DDR1 promoter decreased in COL4A6-knockdown OVCAR-8 cells and increased in COL4A6-overexpressing OVCAR-4 cells (Fig. 3E, lower panel). Furthermore, E2F1 binding to the DDR1 promoter (Fig. 3F, upper panel) was affected by E2F1 knockdown and overexpression in COL4A6-knockdown OVCAR-4 cells; the level of reporter activity (Fig. 3F, right panel) was affected in COL4A6-overexpressing OVCAR-4 cells. Opposite effects were observed when COL4A6-knockdown OVCAR-8 cells were transfected with the E2F1-overexpression plasmid (Fig. 3F, left panel). Evidently, COL4A6-dependent DDR1 induction is mediated by E2F1 binding to the DDR1 promoter.

COL4A6-knockdown OVCAR-8 and COL4A6-overexpressing OVCAR-4 cells were further subjected to ectopic expression and silencing of DDR1, respectively, to clarify whether COL4A6-mediated DDR1 is responsible for cell invasion. The data showed that DDR1, DDR1, and FAK phosphorylations decreased in COL4A6-knockdown OVCAR-8 cells (Fig. 3G) and increased in COL4A6-overexpressing OVCAR-4 cells (Fig. 3H). Notably, the COL4A6-mediated increases in DDR1 expression, DDR1 phosphorylation, and FAK phosphorylation in COL4A6-overexpressing OVCAR-4 cells were reduced by transfection with shRNA against DDR1 (shDDR1, Fig. 3H). Opposite effects were observed when COL4A6-knockdown OVCAR-8 cells were transfected with the DDR1-overexpression plasmid (Fig. 3G). The invasion capacity increased in COL4A6-knockdown OVCAR-8 cells, which was reinstituted by the ectopic expression of DDR1 in the same cells (Fig. 3G, right panel). The opposite effect was observed when COL4A6-overexpressing OVCAR-4 cells were transfected with shDDR1 (Fig. 3H, right panel). This study further explored whether FAK is critical for cell invasion induced by the COL4A6/DDR1 axis. The increased cell invasion ability of COL4A6 cells was abolished after treatment with FAK inhibitor (Fig. 3I). Therefore, COL4A6 may regulate cell invasion through the E2F1/DDR1/FAK pathway.

### COL4A6 increases the levels of phosphorylated DDR1 and FAK in ovarian cancer cells through the stabilization of SHC1

**Fig. 4:**
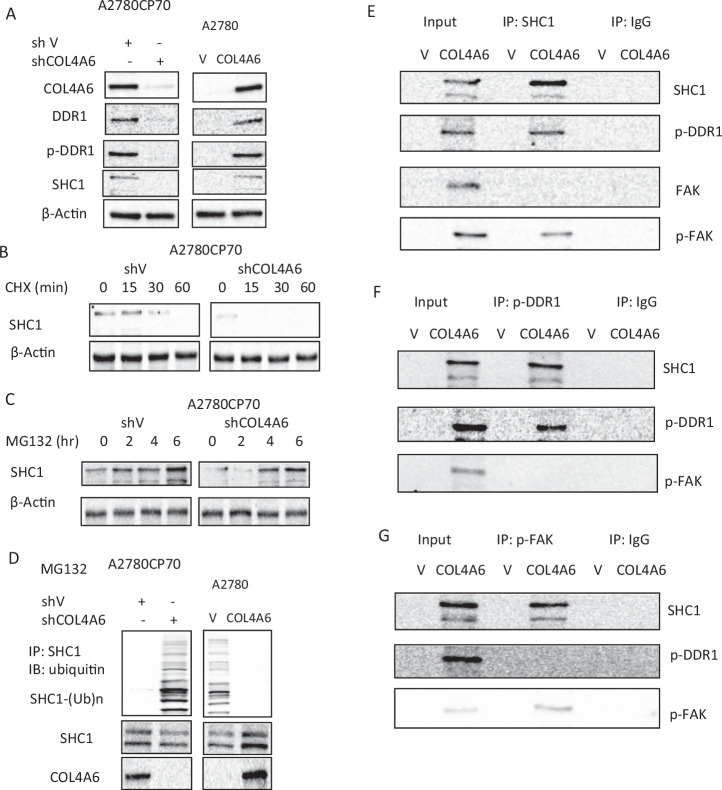
COL4A6 increases phosphorylated DDR1 and FAK levels in ovarian cancer cells through the stabilization of SHC1. **A** COL4A6, DDR1, p-DDR1, and SHC1 protein expression in A2780CP70 cells transfected with shCOL4A6 (3 µg) and A2780 cells transfected with COL4A6 expression plasmid (3 µg) were evaluated by western blotting. β-actin was used as a protein loading control. **B** A2780CP70 cells transfected with shCOL4A6 (3 µg) were incubated with CHX (20 mg/mL) for the indicated times and analyzed by western blotting. β-actin was used as a protein loading control. **C** A2780CP70 cells transfected with shCOL4A6 (3 µg) were treated with MG132 (10 μM) for the indicated times and analyzed by western blotting. β-actin was used as a protein loading control. **D** A2780CP70 cells transfected with shCOL4A6 (3 µg) were treated with MG132 for 6 h; subsequently, the cell lysates were immunoprecipitated with anti-SHC1 antibodies. The immunoprecipitates (IPs) were analyzed by western blotting using an anti-ubiquitin antibody. SHC-1 and COL4A6 protein expressions were evaluated by western blotting. **E**–**G** A2780 cells were transfected with COL4A6 expression plasmid (3 µg) for 48 h, and the cell lysates were immunoprecipitated with anti-SHC1, anti-p-DDR1, anti-p-FAK, and anti-IgG antibodies, and the IPs were analyzed by western blotting.

As mentioned earlier, DDR1 phosphorylation levels varied with the changes in COL4A6 overexpression or COL4A6 silencing (Fig. 3E, G, H). The Src homolog and collagen homolog 1 (SHC1) have a phosphotyrosine-binding (PTB) domain that binds phosphotyrosine residues, including phospho-Tyr-513 of DDR1 [20]. The expressions of DDR1, p-DDR1, and SHC1 were examined, and their expressions were inhibited in shCOL4A6 cells and increased upon COL4A6 overexpression (Fig. 4A). SHC1 protein stability is enhanced by COL4A6. A2780CP70 cells transfected with shCOL4A6 or shcontrol were treated with CHX, an inhibitor of protein synthesis. Western blotting showed that SHC1 protein was degraded more rapidly in shCOL4A6-transfected cells than in shcontrol-transfected cells (Fig. 4B). Furthermore, SHC1 expression increased in shCOL4A6 cells after MG132 treatment (Fig. 4C). SHC1 ubiquitination pattern in A2780 cells was more extensive than in A2780CP70 cells and was rescued by exogenous overexpression of COL4A6. Conversely, COL4A6 silencing facilitated SHC1 ubiquitination in A2780CP70 cells (Fig. 4D).

Immunoprecipitation assays were performed to determine whether SHC1 interacted with p-DDR1 in OC cells. Results showed that SHC1 was immunoprecipitated using antibodies against p-DDR1 in COL4A6-overexpressing A2780 cells (Fig. 4E, F). Surprisingly, SHC1 was found to bind to p-FAK (Fig. 4E, G). However, p-FAK did not bind to p-DDR1 (Fig. 4F, G).

### COL4A6 regulates cell sensitivity to cisplatin via the DDR1/NF-κB axis

**Fig. 5:**
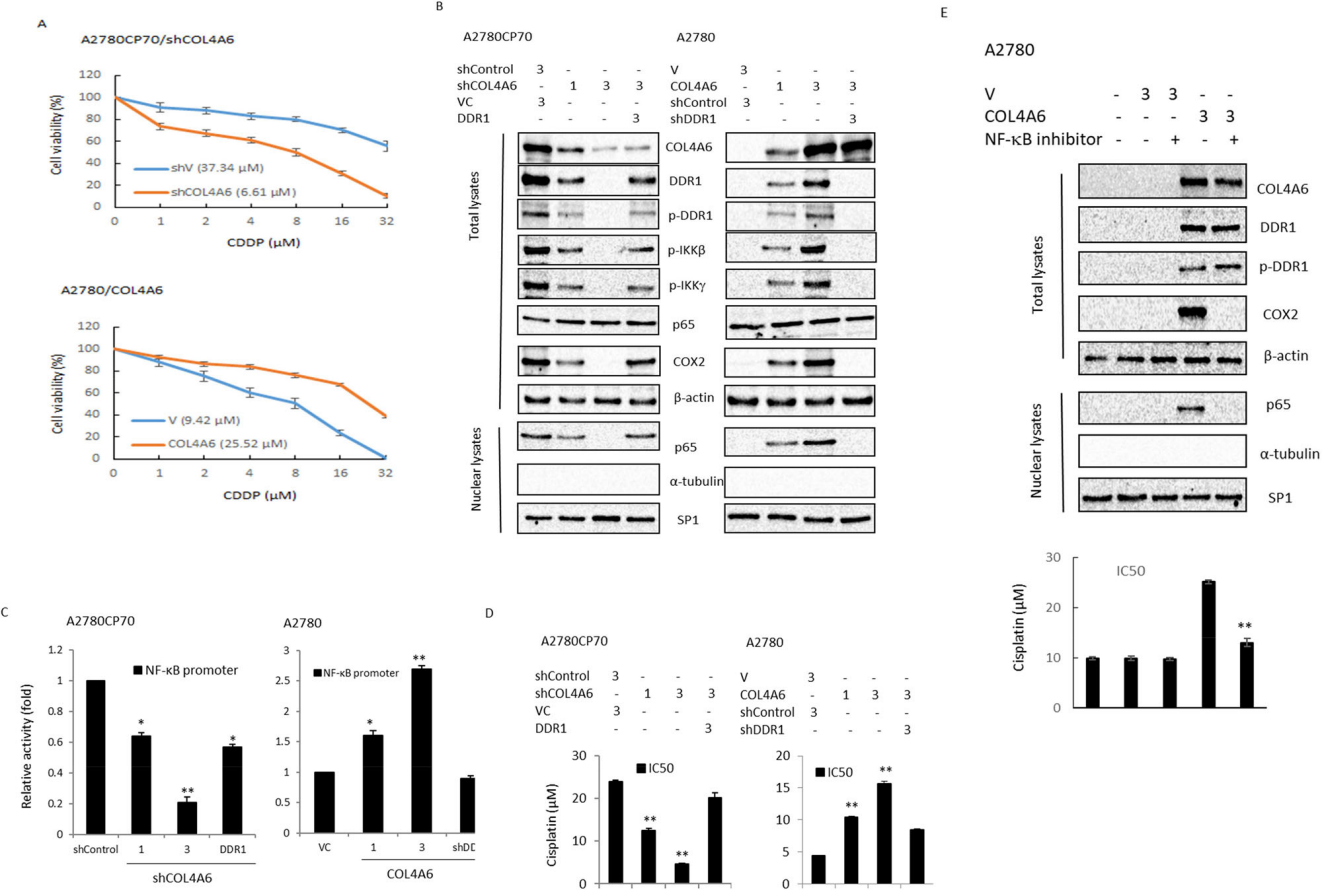
COL4A6 regulates cell sensitivity to cisplatin via the DDR1/NF-κB axis. **A** Upper panel: A2780CP70 cells were transfected with COL4A6 knockdown plasmid. After 48 h, the cells were seeded into 96-well plates and treated with various concentrations of cisplatin for 48 h; subsequently, cell sensitivity to cisplatin was measured using the MTT assay. Lower panel: A2780 cells were transfected with COL4A6-expression plasmid. After 48 h, the cells were seeded into a 96-well plate and treated with various concentrations of cisplatin for 48 h; subsequently, cell sensitivity to cisplatin was measured using the MTT assay. All experiments were performed in triplicate. *P*-values were determined using the Student’s *t*-test. ^*^P < 0.05 or ^**^P < 0.005^,^ relative to control cells treated with shControl or V. **B** A2780CP70 cells were transfected with COL4A6 knockdown plasmid and a DDR1 cDNA plasmid, and A2780 cells were transfected with a COL4A6 cDNA plasmid and DDR1 knockdown plasmid. COL4A6, DDR1, p-DDR1, p-IKKβ, p-IKKγ, p65, COX2, and the nuclear p65 fraction in whole lysates of both types of cells were evaluated by western blotting. β-actin and SP1 were detected as loading controls for whole-cell lysates and nuclear fractions, respectively. **C** A2780CP70 cells were transfected with different quantities of a COL4A6 knockdown plasmid (1 and 3 µg) and a DDR1 cDNA plasmid (3 µg), and A2780 cells were transfected with different quantities of a COL4A6 plasmid (1 and 3 µg) and DDR1 knockdown plasmid (3 µg). Luciferase activities were measured and normalized to Renilla luciferase activities. All experiments were performed in triplicate. *P*-values were determined using the Student’s *t*-test. ^*^P < 0.05 or ^**^P < 0.005, relative to control cells treated with shControl or V. **D** A2780CP70 cells were transfected with different quantities of a COL4A6 knockdown plasmid (1 and 3 µg) and a DDR1 cDNA plasmid (3 µg), and A2780 cells were transfected with different quantities of a COL4A6 plasmid (1 and 3 µg) and DDR1 knockdown plasmid (3 µg). After 48 h, the cells were seeded into a 96-well plate and treated with various concentrations of cisplatin for 48 h; subsequently, cell sensitivity to anticancer drugs was measured using the MTT assay. All experiments were performed in triplicate. *P*-values were determined using the Student’s *t*-test. ^*^P < 0.05 or ^**^P < 0.005, relative *t*o control cells treated with shControl or V. **E** Upper panel: western blotting was performed to evaluate COL4A6, DDR1, p-DDR1, and COX2, and the nuclear p65 fraction in whole lysates of both types of cells was evaluated by western blotting. β-actin and SP1 served as loading controls for whole-cell lysates and nuclear fractions, respectively, after treatment of A2780-COL4A6 expression cells for 24 h with an NF-κB inhibitor. β-actin was the loading control. Lower panel: After 48 h, the cells were seeded into a 96-well plate and treated with various concentrations of cisplatin for 48 h; subsequently, cell sensitivity to anticancer drugs was measured using the MTT assay. All experiments were performed in triplicate. *P* values were determined using the Student’s *t*-test. ^*^P < 0.05 or ^**^P < 0.005, relative to control cells treated with shControl or V.

To examine whether COL4A6 confers resistance to cisplatin, a siRNA specific for the *COL4A6* gene (shCOL4A6) was introduced into cisplatin-resistant A2780CP70 and OVCAR-8 cells, and a *COL4A6* cDNA plasmid was introduced into COL4A6-low expressing cisplatin-sensitive A2780 and OVCAR-4 cells to induce its overexpression. The IC50 of cisplatin was lower in COL4A6-knockdown A2780CP70 and OVCAR-8 cells than in shcontrol cells (Fig. 5A, upper panel for A2780CP70; Supplementary Figure 1A, upper panel for OVCAR-8). Conversely, the sensitivity of COL4A6-overexpressing A2780 and OVCAR-4 cells to cisplatin was lower than that of the vector control cells (V) (Fig. 5A, lower panel for A2780; Supplementary Figure 1A, lower panel for OVCAR-4). Collectively, these data demonstrate that COL4A6 is involved in cisplatin responsiveness regulation.

Increased DDR1 expression confers chemoresistance to ovarian and lung cancers [21, 22]. A previous study demonstrated that DDR1 played an important role in NF-κB-dependent chemoresistance [23]. COL4A6 depletion via RNA interference was found to reduce NF-κB translocation, NF-κB promoter activity, total DDR1 expression, and DDR1 phosphorylation in high COL4A6-expressing A2780CP70 cells (Fig. 5B, C, left panel) and OVCAR-8 (Supplementary Figure 1B and 1C, left panel). Conversely, COL4A6 overexpression increased NF-κB translocation and NF-κB promoter activity by increasing total DDR1 expression and DDR1 phosphorylation expression in low COL4A6-expressing A2780 cells (Fig. 5B, C, right panel) and OVCAR-4 (Supplementary Figure 1B and 1C, right panel). Notably, the COL4A6-mediated increase in total DDR1 expression, DDR1 phosphorylation, nuclear p65 levels, and NF-κB reporter activity in COL4A6-overexpressing A2780 (shDDR1, Fig. 5B, C, right panel) and OVCAR-4 (Supplementary Figure 1B and 1C, right panel) was reduced by transfection with shRNA against DDR1. Opposite effects were observed when COL4A6-knockdown A2780CP70 (Fig. 5B, C, left panel) and OVCAR-8 (Supplementary Figure 1B and 1C, left panel) were transfected with a DDR1-overexpression plasmid. Chemosensitivity to cisplatin increased in COL4A6-knockdown A2780CP70 cells, which was reinstituted by the ectopic expression of DDR1 in the same cells (Fig. 5D, left panel). The opposite effect was observed when COL4A6-overexpressing A2780 cells were transfected with shDDR1 (Fig. 5D, right panel). Whether NF-κB was critical for chemoresistance induced by the COL4A6/DDR1 axis was explored. The increased cell resistance to cisplatin by COL4A6 was abolished after treatment with an NF-κB inhibitor (Fig. 5E). Therefore, COL4A6 regulates cisplatin sensitivity via the DDR1/NF-κB axis.

### DDR1-IN-1 regulates cell sensitivity to cisplatin and cell invasiveness via COL4A6 inhibition

**Fig. 6:**
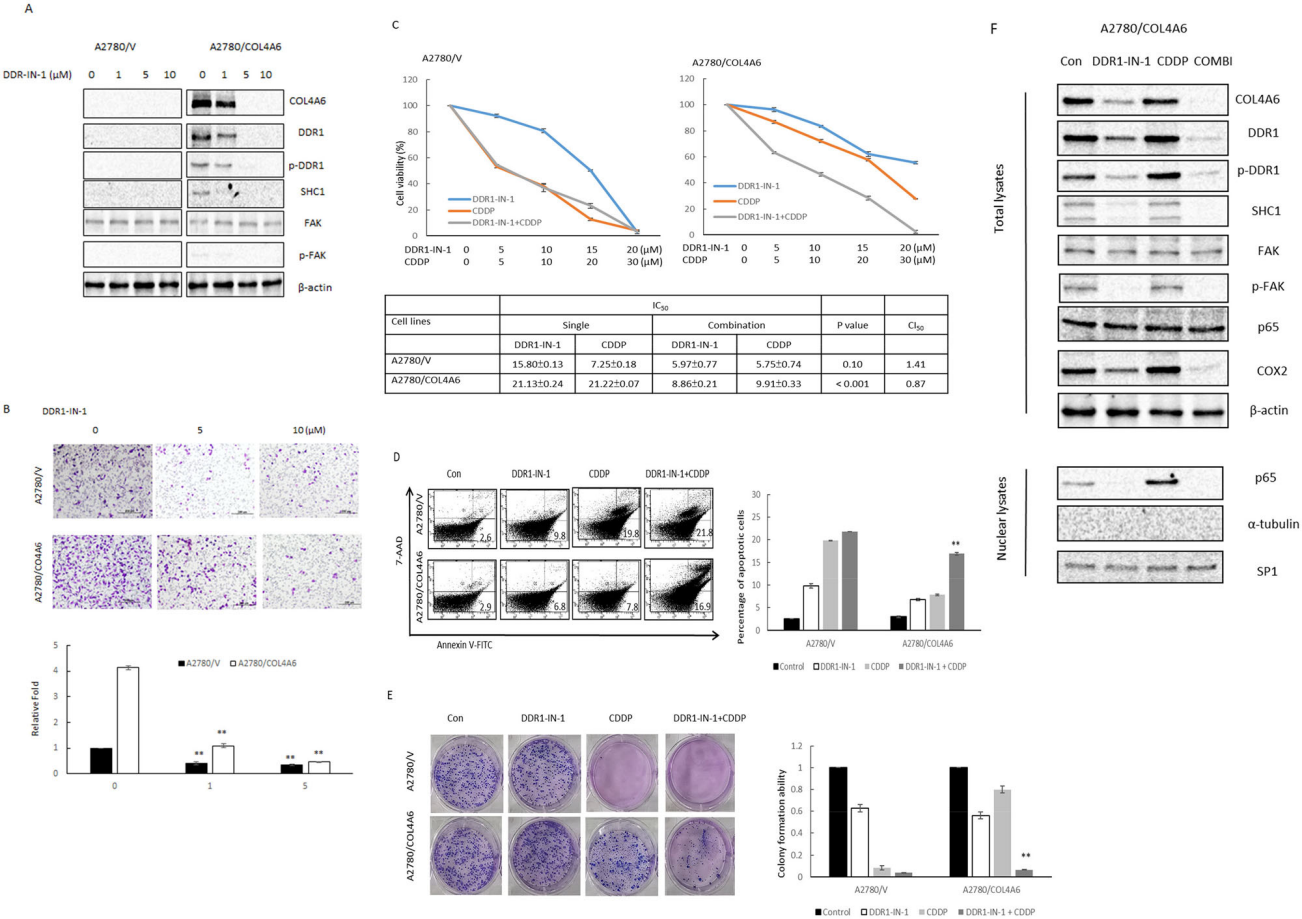
DDR1-IN-1 increases cell sensitivity to cisplatin and synergizes with cisplatin-mediated cell apoptosis via COL4A6 inhibition. **A** Protein expression levels of COL4A6, DDR1, p-DDR1, SHC1, FAK, and p-FAK in A2780/V and A2780/COL4A6 cells treated with different concentrations of DDR1-IN-1 for 24 h were evaluated by western blotting. β-actin was used as an internal loading control. All experiments were performed in triplicate. **B** Invasion activity in vitro of A2780/V and A2780/COL4A6 cells after treatment with different concentrations of DDR1-IN-1 for 24 h. All data represent the means ± standard deviations of three separate experiments. ^*^P < 0.05 and ^**^P < 0.005^,^ SC66 versus control. **C** Ovarian cancer cells were treated with different concentrations of cisplatin (0–30 μM) or combined with different concentrations of DDR1-IN-1 (0–20 μM) for 48 h. Each combination was tested with n = 5 replicates. After 48 h, cell viability was assessed by MTT assays. All experiments were performed in triplicate. The IC_50_ values of each agent alone or in combination treatments and CI values of the DDR1-IN-1 + CDDP combinations. *P*-value between the IC_50_ values of single versus combination treatment. **D** A2780/V and A2780/COL4A6 cells were treated for 24 h with 5 μM DDR1-IN-1 alone or with the addition of cisplatin (10 μM) indicated. The percentage of apoptotic cells was determined by annexin V and 7-AAD staining. The mean ± standard deviation for three independent experiments is shown. ^**^P < 0.005, DDR1-IN-1 ^+^ CDDP versus CDDP. **E** Colony formation assay. A2780/V and A2780/COL4A6 cells were treated with 5 μM DDR1-IN-1 alone or with the addition of CDDP (10 μM) as indicated for 14 days. After treatment, the cells were stained with crystal violet. The mean ± standard deviation for three independent experiments is shown. ^**^P < 0.005, DDR1-IN-1 + CDDP versus CDDP. **F** Western blotting was performed to evaluate COL4A6, DDR1, p-DDR1, SHC1, FAK, p-FAK, p65, COX2, and the nuclear p65 fraction in whole lysates of both types of A2780-COL4A6 expression cells after treatment with 5 μM DDR1-IN-1 alone or with the addition of CDDP (10 μM) for 24 h. β-actin and SP1 served as loading controls for whole-cell lysates and nuclear fractions, respectively. β-actin was the loading control.

Inhibition of the DDR signaling pathway can reduce metastasis, dissemination, or reactivation and prevent disease relapse [24, 25]. DDR1-IN-1 is a potent and selective DDR1 receptor tyrosine kinase inhibitor [26, 27]. The results indicate that DDR-IN-1 suppresses the elevated expression levels of COL4A6, total DDR1 expression, DDR1 phosphorylation, SHC1, and FAK phosphorylations in A2780/COL4A6 cells (Fig. 6A). Next, it was examined whether DDR1-IN-1 treatment decreases COL4A6-mediated cell invasiveness via p-DDR1 inhibition. As shown in Fig. 6B, the cell invasion ability increased in A2780/COL4A6 cells compared with A2780/V cells, and the increased invasiveness was inhibited by adding DDR1-IN-1.

Whether DDR1-IN-1 could overcome COL4A6-mediated chemoresistance in OC cells was further investigated. The CI showed the synergistic cytotoxicity of DDR1-IN-1 and cisplatin in A2780/COL4A6 cells (CI_50_ = 0.87, *P* < 0.001 for DDR1-IN-1 + CDDP vs. CDDP, Fig. 6C). In contrast, synergistic cytotoxicity was not observed in the A2780/V cells (Fig. 6C). Representative apoptotic profiles further indicated that the combination treatment of A2780/COL4A6 cells with DDR1-IN-1 and cisplatin increased the apoptotic cell population (Fig. 6D). Consistent with these results, the combined treatment of chemoresistant A2780/COL4A6 cells with DDR1-IN-1 and cisplatin resulted in a significantly stronger inhibitory effect on colony formation than treatment with DDR1-IN-1 or cisplatin alone (Fig. 6E). Western blotting analysis further showed that the expression levels of COL4A6, total DDR1 expression, DDR1 phosphorylation, FAK phosphorylation, COX2, and nuclear p65 were slightly increased in A2780/COL4A6 cells treated with cisplatin, and this elevation was inhibited by combined treatment with DDR1-IN-1 and cisplatin (Fig. 6F). Altogether, these results demonstrate that DDR1-IN-1 treatment sensitizes cells to cisplatin treatment and promotes apoptosis by inhibiting COL4A6 activation.

### DDR1-IN-1 enhances anticancer drug therapy in mouse

To determine whether the DDR1-IN-1 antibody could suppress COL4A6-mediated tumor growth in vivo, mice were injected intraperitoneally with 1 × 10^6^ A2780/COL4A6 cells and then treated with an intraperitoneal injection of DDR1-IN-1 with or without cisplatin. Western blotting confirmed that COL4A6 expression was increased in the A2780 cell clone, stably expressing COL4A6 compared with A2780/V cells (Supplementary Fig. S3A). The intraperitoneal tumors spread rapidly to the intestine, ovary, kidney, and liver (Supplementary Fig. S3B), which was evident in the groups inoculated with COL4A6-expressing A2780 cancer cells and sacrificed on day 22. Notably, ascites and tumors on the diaphragm were evident in mice inoculated with A2780/COL4A6 cells that were sacrificed on day 41 (Supplementary Fig. S3C). In contrast, tumor formation inside the peritoneum was not observed in A2780/V inoculated mice sacrificed on day 41 (Supplementary Fig. S3D). These results suggest that COL4A6 plays an important role in tumor formation and spread.

**Fig. 7:**
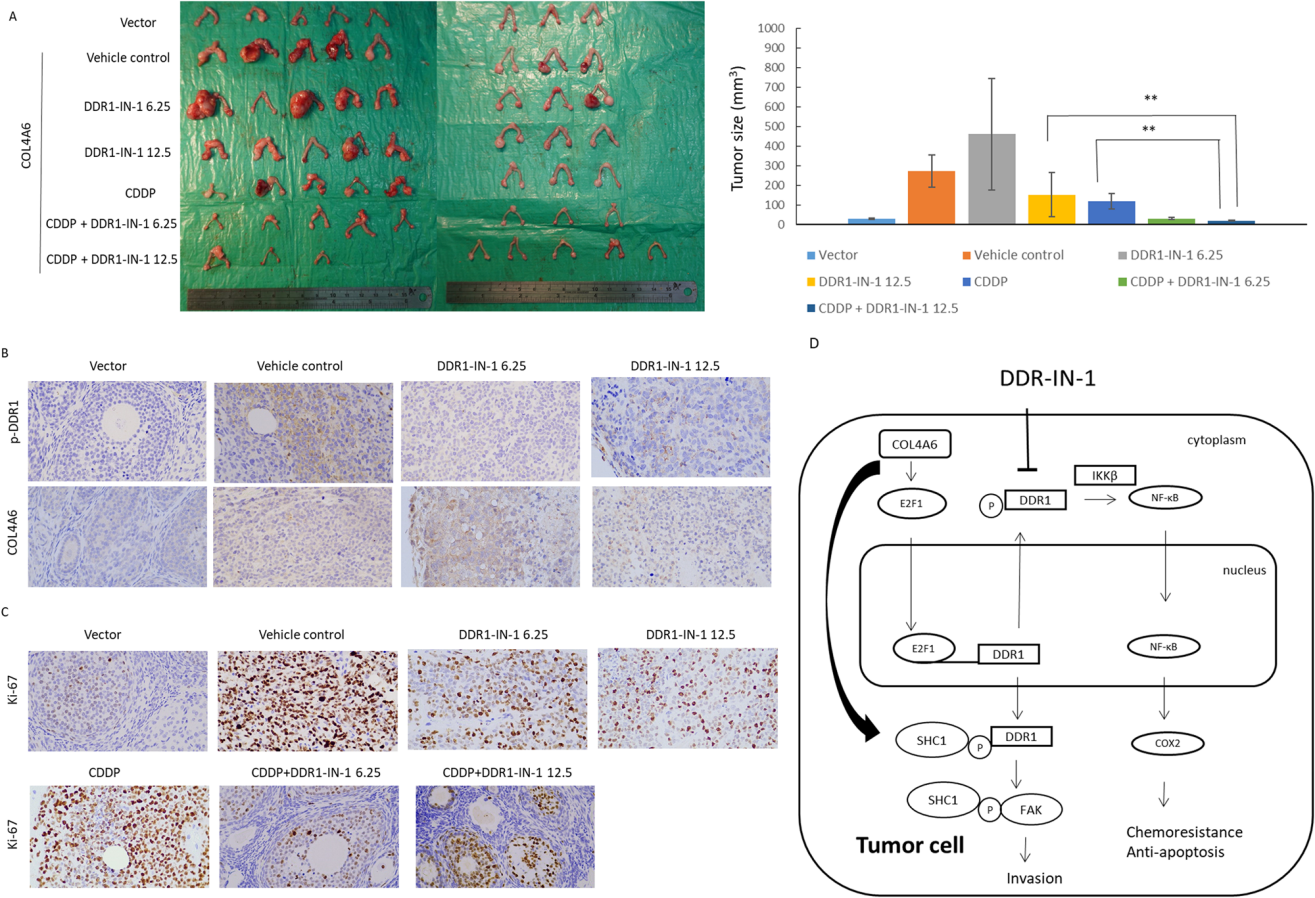
DDR1-IN-1 combined with cisplatin shows synergistic efficacy in mouse xenografts. **A** A2780/COL4A6 ovarian cancer xenografts treated with only DDR1-IN-1 at doses of 6.25 mg/kg and 12.5 mg/kg or combined with 2 mg/kg cisplatin. ^**^P < 0.005, on day 28, DDR1-IN-1 12.5 mg/kg + CDDP 2 mg/kg vs. CDDP alone (*P* < 0.001) or varying doses of DDR1-IN-1 (6.25 mg/kg, *P* < 0.001; 12.5 mg/kg, *P* < 0.001). **B** Representative immunohistochemical photos of p-DDR1 and COL4A6 in ovarian tumor samples from mice treated with DDR1-IN-1 or vehicle controls. **C** Representative immunohistochemical photos of Ki-67 in ovarian tumor samples from mice treated with DDR1-IN-1 or vehicle controls. **D** A model illustrating the hypothetical role of COL4A6 regulation in ovarian cancer cells.

When compared with the treatment vehicle controls, a single treatment with 2 mg/kg cisplatin (*P* = 0.01) or varying doses of DDR1-IN-1 (6.25 mg/kg, *P* = 0.72; 12.5 mg/kg, *P* = 0.02) inhibited tumor growth on day 28 (Fig. 7A). Furthermore, tumor size was significantly reduced in mice treated with 12.5 mg/kg DDR1-IN-1 plus 2 mg/kg cisplatin compared with that in mice treated with either 2 mg/kg cisplatin alone (*P* < 0.001) or 12.5 mg/kg DDR1-IN-1 alone (12.5 mg/kg, *P* < 0.001).

The p-DDR1 and COL4A6 protein levels decreased in the cancerous tissues of mice treated with DDR1-IN-1 compared with those of the vehicle controls (Fig. 7B). Ki-67 expression was significantly reduced in mice treated with 12.5 mg/kg DDR1-IN-1 + 2 mg/kg cisplatin compared to mice treated with 2 mg/kg cisplatin alone (*P* < 0.001, Fig. 7C). The body weights of animals receiving cisplatin or DDR1-IN-1 alone or in combination remained relatively unchanged, suggesting a negligible level of toxicity caused by the treatments (data not shown).

## Discussion

This study’s principal finding was that COL4A6 played an important role in the regulation of cell aggressiveness, cell proliferation, tumor formation, and chemoresistance and that COL4A6 expression acted as a predictor of poor clinical outcomes in patients with EOC. COL4A6 may promote cell aggressiveness via the DDR1/FAK axis, and the E2F1 binding site on the DDR1 promoter is the major determinant of COL4A6-dependent DDR1 activation. COL4A6 regulated chemoresistance via the DDR1/NF-κB axis. Additionally, high COL4A6 mRNA levels were associated with extrapelvic spread, disease progression, poor PFS, and OS in patients with OC. Based on these findings, we propose that COL4A6 increases cancer chemoresistance (Fig. 7D) and may serve as a therapeutic biomarker in EOC.

A previous study indicated that five collagen subunits (COL1A1, COL4A1, COL4A2, COL4A5, and COL4A6) were upregulated in oral tumor tissues compared with their normal counterparts [28]. Among these, COL4A5 and COL4A6 were significantly co-overexpressed with DDR1 in 40 oral tumor tissues. In a cell model, COL4 increased DDR1 mRNA and protein levels in primary or immortalized oral keratinocytes [28]. COL4A6 regulated DDR1 transcription by increasing the binding of E2F1 to the DDR1 promoter (Fig. 3). Tissue collagen may be a key driver of DDR1 expression in cancer cells. Further studies are required to explore the mechanism by which E2F1 is regulated by COL4A6.

Collagens are a group of ECM proteins crucial for tissue organization and structural integrity. Under pathological conditions, abnormal collagen deposition contributes to tissue fibrosis and cancer progression. These findings suggest that increased COL4A6 expression may influence tumor-microenvironment interactions, potentially enhancing cancer cell aggressiveness. COL4A6, DDR1, and p-DDR1 expression levels were elevated in A2780 cells cultured in a medium conditioned by A2780CP70 cells (Supplementary Figure 4). Factors secreted by A2780CP70 cells, possibly including COL4A6, can influence COL4A6 expression and signaling in A2780 cells. Further investigation is needed to determine whether exogenous COL4A6 alone is sufficient to drive these effects. Additionally, studies should explore how COL4A6 interacts with the tumor microenvironment and identify potential therapeutic strategies to modulate its expression or stromal interactions to overcome chemoresistance.

DDR1 is a receptor tyrosine kinase activated by various types of collagen and plays a role in cell attachment, migration, survival, and proliferation. Abnormal DDR1 expression is detected in a range of solid tumors (including breast, ovarian, cervical, liver, gastric, colorectal, lung, and brain) [29–35] and is associated with aggressive metastatic tumors (including breast and ovarian) and poor prognosis [24, 36–38]. During tumorigenesis, abnormal activation of DDR1 leads to invasion and metastasis via the dysregulation of cell adhesion, migration, proliferation, cytokine secretion, ECM remodeling, and angiogenesis [39–41]. DDR1 expression is associated with decreased OS in patients with metastatic colorectal cancer, and DDR1 phosphorylation is strongly increased in the corresponding metastatic lesions [42, 43], indicating that COL4A6 promotes cell invasion via the E2F1/DDR1/FAK axis. These findings are consistent with previous data showing that DDR1 facilitates the migration and invasion of breast cancer cells via FAK signaling pathway activation [17], which is also one of the main downstream effectors of COL4A6 [18].

Moreover, DDR1 expression reduces the sensitivity to chemotherapy, which may lead to cancer recurrence [43–46]. Increased DDR1 expression confers chemoresistance to ovarian and lung cancers [21, 22]. DDR1 serves as both a potential biomarker and molecular target in advanced OC [47]. In this study, COL4A6 conferred chemoresistance via DDR1-mediated NF-κB expression in OC cells. NF-κB has a critical contribution to the resistance of various cancers to conventional therapies, such as chemotherapy and radiotherapy [48]. Furthermore, the NF‑κB pathway can be targeted as a potential regulator of immune checkpoints in cancer immunotherapy [48]. The RNA sequencing data pointed out gene sets involving signaling receptor activator activity (DDR1), cytokine activity (p65), and regulation of immune response in A2780CP70 cells with COL4A6 knockdown (data not shown). COL4A6 may modulate the response to immunotherapy. Further studies are warranted to explore the precise mechanisms underlying COL4A6 effect on the immune microenvironment and response to immunotherapy in EOC. KEGG enrichment analysis revealed that COL4A6 knockdown in A2780CP70 cells altered the PI3K-Akt signaling pathway and led to decreased expression of inhibitors of apoptosis proteins (IAPs) (data not shown). Given that IAPs are known mediators of the PI3K/Akt pathway, COL4A6 may contribute to cisplatin resistance by promoting cell survival through apoptotic pathway modulation.

DDR1 comprises three regions: an extracellular ligand-binding domain, a transmembrane region, and an intracellular region containing a kinase domain. Upon activation, DDR1 autophosphorylates multiple intracellular tyrosine residues. Phosphorylation of DDR1 recruits cytoplasmic signaling adaptors containing SHC1 or PTB motifs, which in turn assemble more signaling molecules to execute various biological processes involved in cell migration, differentiation, and ECM remodeling [49, 50]. Here, COL4A6 could increase phosphorylated DDR1 in OC cells by stabilizing SHC1. Further evidence for these observations was provided by the reduced SHC1 protein expression observed in COL4A6-knockdown cells, the marked increase in SHC1 in the presence of the 26S proteasome inhibitor MG132, and SHC1 proteasomal degradation observed in COL4A6 defects. Phosphorylated DDR1 levels in OC cells were increased by COL4A6 via increased binding between SHC1 and p-DDR1 (Fig. 4E, F). Collectively, COL4A6 overexpression prevents SHC1 degradation, increases SHC1 protein stability, and promotes SHC1 interaction with p-DDR1. To the best of our knowledge, this is the first study to report the binding activity of SHC1-p-DDR1 in OC cells.

COL4A6 was found to play an important role in EOC. COL4A1 promotes cell aggressiveness by upregulating the DDR1/FAK axis and predicts a poor clinical outcome in patients with OC. Moreover, COL4A6 promoted cancer cell sensitivity to cisplatin via DDR1/NF-κB pathway activation. A previous report indicated that DDR1 inhibitors were currently under clinical testing for multiple indications (merestinib against metastatic breast cancer [NCT03292536], merestinib against HR+ or HER2– breast cancers [NCT02791334]) [51]. Another DDR1 inhibitor, sitravatinib, induces tumor immune landscape changes that enhance the efficacy of immune checkpoint blockade in refractory cancer models [52]. DDR1-IN-1 is a potent and selective DDR1 receptor tyrosine kinase inhibitor [26, 27]. A previous report indicated that DDR1-IN-1 suppressed oral cancer cell growth and clonogenicity and inhibited the invasion of TW2.6 cells in vivo [26]. DDR1-IN-1 combined with radiochemotherapy increases glioblastoma cell sensitivity to therapy [53]. In this study, DDR1-IN-1 treatment sensitized cells to cisplatin and promoted apoptosis by inhibiting COL4A6 activation.

Collagen IV is a major ECM protein in tumors, particularly within the stroma, where it provides structural support and regulates tumor progression. Given its stromal localization, targeting COL4A6 with antibody-drug conjugates (ADCs) or other therapeutic strategies may offer a novel approach to overcoming chemoresistance, as has been explored in pancreatic cancer [54].

While Type IV collagen has been linked to the tumor stroma in pancreatic cancer, COL4A6’s specific role in OC progression, particularly in advanced stages, remains poorly understood. Further research is needed to determine whether COL4A6 influences stromal remodeling, metastasis, and chemoresistance and if it can serve as a biomarker or therapeutic target. A deeper understanding of these mechanisms may lead to novel treatment strategies aimed at disrupting the tumor microenvironment and improving chemotherapy response in advanced OC.

## Conclusion

COL4A6 may promote tumor aggressiveness and chemoresistance via the E2F/DDR1 axis, and COL4A6 expression can predict clinical outcomes in patients with OC. DDR1 should be targeted in patients with COL4A6-positive tumors.

## Materials and Methods

The details of quantitative reverse transcriptase polymerase chain reaction (qRT-PCR), cells and media, plasmid constructs and transfection, western blotting analysis, cell fractionation, antibodies and reagents, cell proliferation, transwell invasion assay, calculation of half maximal inhibitory concentration and combination index analysis, plasmid construction and site-directed mutagenesis, luciferase reporter assays, chromatin immunoprecipitation assays, annexin V-binding assay for apoptosis and colony formation assay are provided in the Supplementary Information.

### Study population

This study adhered to the tenets of the Declaration of Helsinki, and the research protocol was approved by the Institutional Review Boards of National Cheng Kung University Hospital (No. B-ER-107-396) and Chi Mei Medical Center (10808-L03). Informed consent was obtained from all patients. Patients with EOC who underwent primary cytoreductive surgery at the National Cheng Kung University Hospital and Chi Mei Medical Center, Liouying, and who received front-line postoperative platinum-based chemotherapy between January 1, 2010, and December 31, 2016, were considered eligible for study participation.

Patients’ medical records were reviewed, and their clinical characteristics and information regarding cancer stage, front-line chemotherapy, response to chemotherapy, and treatment outcomes were collected. Their follow-up records from May 20, 2022, were reviewed. OS was calculated according to the date of diagnosis, whereas PFS and progression-free interval (PFI) were determined based on the date of last contact or progression following front-line chemotherapy. Patients with EOC with PFIs ≤ or >6 months were divided into “resistant” and “sensitive” to platinum-based chemotherapy groups, respectively. The post hoc power analysis showed that assuming a moderate effect size (Cohen’s d = 0.5), a significance level (α) of 0.05, and a power (1–β) of 0.80, the required sample size would be approximately 128 patients. Therefore, our current sample size (n = 160) is considered sufficient to detect clinically meaningful differences with adequate statistical power.

### Animal model

All animal procedures were reviewed and approved by the Institutional Animal Care and Use Committee of Chi Mei Medical Center. In the pretest experiment, female 6-week-old NOD-SCID mice (National Cheng University Animal Center) were intraperitoneally injected with 1 × 10^6^ (100 μL) A2780/V or A2780/COL4A6 cells (*n* = 2 per group). Humane sacrifice methods were employed when intra-abdominal tumors caused abnormal expansion of the abdominal cavity and difficulty in breathing, eating, drinking, or moving.

For DDR1-IN-1 and cisplatin treatment, to reject the null hypothesis that the population means of the experimental and control groups were equal, the sample size for these groups was required to be sufficient, with a power of 0.8 and a type I error of 0.01. The number of animals required was estimated using PS: Power and Sample Size Calculation, version 3.1.2, developed by William D. Dupont and Walton D. Plummer Jr. (Vanderbilt University, Nashville, TN, USA). The number of mice required to assess tumor volume was estimated to be at least eight each for the control and experimental groups. Female 6-week-old NOD-SCID mice (National Cheng University Animal Center) were intraperitoneally injected with 1 × 10^6^ (100 μL) A2780/V or A2780/COL4A6 cells. After 7 days, the mice were intraperitoneally injected with (1) A2780/V (saline, vehicle control), (2) A2780/COL4A6 (vehicle control), (3) A2780/COL4A6 (DDR1-IN-1 6.25 mg/kg), (4) A2780/COL4A6 (DDR1-IN-1 12.5 mg/kg), (5) A2780/COL4A6 (cisplatin, CDDP 2 mg/kg), (6) A2780/COL4A6 (CDDP + DDR1-IN-1 6.25 mg/kg), and (7) A2780/COL4A6 (CDDP + DDR1-IN-1 12.5 mg/kg). Animals in each group received 100 μL of saline, cisplatin, or DDR1-IN-1 by intraperitoneal injection. DDR1-IN-1 was generally formulated as a suspension in PEG300/TWEEN80/saline. The treatment frequencies were once per day for DDR1-IN-1 and once per 3 days for cisplatin. After 28 days, the mice were sacrificed by CO_2_ inhalation, and the tumor tissues were excised. Tumor imaging and body weight were determined, as previously described [55]. Tumor volumes were calculated as length (mm) × width (mm) × height (mm) × 0.52. Animal studies involving tumor volume measurement were usually performed with one control per subject [55].

For histopathology and immunohistochemistry, the tumors were removed, fixed in 10% formalin, embedded in paraffin, and sectioned (4 μm). Paraffin sections of the tumors were stained with hematoxylin and eosin, and a pathologist interpreted the extent of cancer involvement in each organ. Immunohistochemistry was performed using anti-Ki-67 (61-0078) from GeneMed (South San Francisco, CA, USA). The results were scored by multiplying the percentage of positive cells with the intensity. The percentage of positive cells was quantified by counting at least 100 cells in each sample under a light microscope (×400) for each sample. Staining intensity was divided into three classes: weak (1), moderate (2), and strong (3).

### Statistical analyses

Data were analyzed using the SPSS statistical software (version 21.0, IBM Corp., Armonk, NY, USA). Categorical variables are presented as frequencies and percentages and were analyzed using the chi-squared or Fisher’s exact test. Continuous variables are expressed as the means ± standard deviations or as the medians ± interquartile ranges. Interval variables were analyzed using the Student’s t-test or Mann–Whitney U test. The cut-off values obtained based on the receiver operating characteristic curve for COL4A6 were optimized for diagnostic sensitivity and specificity in predicting cancer progression or death. Survival was estimated using the Kaplan–Meier method and compared using the log-rank test. Two-sided *P*-values < 0.05 were considered statistically significant. Cox proportional hazard models were used to estimate the hazard ratios (HRs) and 95% confidence intervals (CIs).

## Supplementary information

Supplementary information

Supplementary figure 1

Supplementary figure 2

Supplementary figure 3

Supplementary figure 4

Supplementary figure 5

## Data Availability

The data supporting the findings of this study can be found in the article, or available from the corresponding author upon reasonable request.
