# Supplementary material for: Collagen type IV alpha 6 promotes tumor progression and chemoresistance in ovarian cancer by activating the discoidin domain receptor 1 pathway
Source: Oncogenesis. 2025 Jul 2;14(1):23. doi: 10.1038/s41389-025-00565-2 (PMC12222940; doi:10.1038/s41389-025-00565-2)
Supplement: Supplementary file 1 — Supplementary information [file 41389_2025_565_MOESM1_ESM.docx]

**Collagen type IV alpha 6 promotes tumor progression and chemoresistance in ovarian cancer by activating the discoidin domain receptor 1 pathway**

Yi-Hui Wu^1,2^, Pei-Ying Wu^3^, Yu-Fang Huang^3^, Chien-Chin Chen^4,5,6,7^, Soon-Cen Huang^8#^, and Cheng-Yang Chou^3#^

**Materials and Methods**

**Quantitative reverse transcriptase polymerase chain reaction (qRT-PCR)**

The RNA obtained (5 μg) was used as a template for cDNA synthesis reactions, together with random primers and superscript III reverse transcriptase (Applied Biosystems). The resultant cDNA solution (1:20) was used to detect the level of the target gene mRNA using quantitative polymerase chain reaction (qPCR). Accurate quantitation was achieved based on standard curves, which were drawn by serially diluting a known amount of RNA obtained through an *in vitro* transcription reaction and by performing TaqMan qPCR using these dilutions, in addition to using patient samples. Quantitative analysis of the mRNA expression was performed using the Light Cycler® 2.0 System (Roche Diagnostics GmbH). Primers and TaqMan probes used for the analyses were designed using the Primer Express software. The following primers were used: COL4A6 (HS00361494) and glyceraldehyde-3-phosphate dehydrogenase (GAPDH) (HS99999905). No-reverse-transcription (no-RT) control reactions were performed using 100 ng of total RNA derived from each individual sample as a template to ensure that amplification did not occur due to DNA contamination. No signals corresponding to the no-RT control were detected. Target gene mRNA expression was assessed by real-time RT-PCR. *GAPDH* was used as an internal control for RNA quality. All quantitative analyses were performed in duplicate to assess the consistency of results. The relative expression levels of target genes, normalized to those of *GAPDH*, were calculated as follows: ΔC_t_ = C_t_(target)–C_t_(*GAPDH*). The ratio of the number of copies of target mRNA to the number of copies of *GAPDH* was calculated as 2^-Ct^ K (K = 10^6^, a constant). Relative fold changes in gene expression were calculated using the comparative 2^−ΔΔCT^ method.

**Cells and media**

OVCAR-4 and OVCAR-8 cell lines were purchased from the National Cancer Institute DTP Tumor Repository Program. ES-2 cells were purchased from the Bioresource Collection and Research Center of the Food Industry Research and Development Institute (Hsinchu, Taiwan). The HAC-2 cells were obtained from the Japanese Collection of Research Bioresources Cell Bank (Osaka, Japan). The A2780 and A2780CP70 cell lines were kindly provided by Dr. Hsu Keng-Fu (Department of Obstetrics and Gynecology, National Cheng Kung University Hospital, College of Medicine, National Cheng Kung University, Tainan, Taiwan). OVCAR-4, OVCAR-8, A2780, and A2780CP70 cells were grown in Roswell Park Memorial Institute-1640 medium supplemented with 10% fetal bovine serum (FBS). ES-2 cells were grown in Mycos 5A medium supplemented with 10% FBS. HAC-2 cells were grown in minimal essential medium supplemented with 15% FBS. These cells were grown at 37 °C in a 5% carbon dioxide atmosphere. The cells were passaged 5–20 times. Routine cell authentication was performed approximately every 6 months using cell morphology monitoring, growth curve analysis, species verification via isoenzymology, karyotyping identity verification via short tandem repeat profiling analysis, and contamination checks. The most recent authentication was conducted in March 2024.

**Plasmid constructs and transfection**

Short hairpin RNAs (shRNAs) directed against human COL4A6 (sc-91179-SH), discoidin domain receptor 1 (DDR1) (sc-35187-SH), E2F1 (sc-29297-SH), and the nontargeting negative control shRNA (control shRNA) were purchased from Santa Cruz Biotechnology (Dallas, TX, USA). COL4A6 (RG212380), DDR1 (RC600061), E2F1 (RG208247), and pCMV6 (PS100001) expression vectors were procured from OriGene. The nuclear factor kappa B (NF-κB) promoter was kindly provided by Prof. Hsiao-Sheng Liu (Department of Microbiology and Immunology, National Cheng Kung University, Tainan, Taiwan). Expression plasmids were transfected into ovarian cancer cells using the HyFect^TM^ DNA transfection reagent, according to the manufacturer’s protocol (Leadgene Biomedical, Taiwan).

**Western blotting analysis**

Proteins were extracted, and equal amounts were separated by 8–15% sodium dodecyl sulfate-polyacrylamide gel electrophoresis (SDS-PAGE), as previously described (1).

**Cell fractionation**

Cells were incubated in extraction buffer A (10 mM Tris-HCL [pH 8.0], 10 mM NaCl, 0.2% NP-40, 2 mM phenylmethylsulfonyl fluoride, 1 mg/mL pepstatin A, 0.2 mM leupeptin, 10 μg/mL aprotinin, 1 mM sodium vanadate, 1 mM nitrophenyl phosphate, and 5 mM benzamidine) for 30 min at 4 °C, followed by centrifugation at 3,000 rpm for 5 min at 4 °C. The supernatant was used as the cytoplasmic extract. The pellet was washed with extraction buffer A, incubated in radioimmunoprecipitation assay buffer for 30 min at 4 °C, and centrifuged at 13,000 rpm for 10 min at 4 °C. The supernatant was used as the nuclear extract. Equal amounts of cytoplasmic and nuclear extracts were resuspended in SDS loading buffer containing 2-mercaptoethanol and boiled for 10 min. Equal amounts of protein were separated by 8–15% SDS-PAGE.

**Antibodies and reagents**

Antibodies against COL4A6 (sc-398655), E2F1 (sc-251), p65 (sc-398442), SP1 (sc-420), and β-actin (sc-47778) were purchased from Santa Cruz Biotechnology (Dallas, TX, USA). Antibodies against phospho-IKKβ (p-IKKβ, Tyr199, GTX55109), phospho-IKKγ (p-IKKγ, Ser31, GTX32282), Src homology and collagen homology 1 (SHC1) (GTX50620), COX2 (GTX60935), and α-tubulin (GTX62882) were obtained from GeneTex (Irvine, CA, USA). Antibodies against focal adhesion kinase (FAK) (AF6397), phospho-FAK (p-FAK, Tyr397, AF3398), and phospho-DDR1 (p-DDR1, Tyr513, and AF2312) were purchased from Affinity Biosciences (Cincinnati, OH, USA). Antibodies against DDR1 (5583), mouse IgG (7076), and rabbit IgG (7074) were purchased from Cell Signaling Technology (Danvers, MA, USA). FAK inhibitor (PF-573228), NF-κB (HY-13453), and DDR1-IN-1 (HY-13979) were purchased from MedChemExpress (Monmouth Junction, NJ, USA). Cisplatin (Fresenius Kabi Oncology, Ltd.) was provided by the Cancer Center of National Cheng Kung University Hospital. MG132 and cycloheximide (CHX) were obtained from Sigma-Aldrich.

**Cell proliferation**

The cell proliferation assay was performed after 24 hours post-transfection. Cells (10^3^/mL) were seeded into a 35-mm dish and cultured for 24, 48, 72, and 96 h, and the cell number at each culture time point was counted to calculate the doubling time.

**Transwell invasion assay**

The Transwell cell invasion assay was performed using polycarbonate membranes with 8-μm pores (Costar, Cambridge, MA, USA). Cells (5×10^4^) were seeded on the membrane of the upper chamber of the Transwell pre-coated with rat collagen I (60 µg/Transwell). Fibronectin in medium (0.6 mL) was added to the lower chamber as a chemoattractant in a 24-h assay at 37 °C under 5% CO_2_. The cells remaining in the upper chamber that did not migrate were removed using a cotton swab. The filters were fixed in 95% ethanol and stained with 0.005% crystal violet for 1 h. Migrated cells were counted using a phase-contrast microscope (Olympus, Lake Success, NY, USA). The mean of 10 contiguous fields represented the cell number. Each experiment was performed in triplicate. The invasive capacity of the cells was normalized to that of the corresponding control. One-sample unpaired Student’s t-test was used to analyze the differences between the normalized invasive capacities determined using three independent experiments and the hypothetical value (set to 1).

**Calculation of half maximal inhibitory concentration and combination index analysis**

Cisplatin (10 mM) was dissolved in distilled water, whereas DDR1-IN-1 (10 mM) stock solutions were prepared in dimethylsulfoxide, stored at −20 °C, and diluted to a final dimethylsulfoxide concentration of < 0.5%. During the experiment, culture medium was used for drug dilution. Cells (1 × 10^4^) were exposed to varying concentrations of cisplatin (0–32 μM) and DDR1-IN-1 (0–20 μM) for 48 h. The *in vitro* cytotoxic effects of these treatments were determined using the 3-(4,5-dimethylthiazol-2-yl)-2,5-diphenyltetrazolium bromide (MTT) assay (at 570 nm) (MTT final concentration, 0.125 mg/mL). Cell viability was expressed as a percentage of the viability of control cells (% of control). The half maximal inhibitory concentration (IC_50_) values were determined from a dose–response curve of percent growth inhibition against the test concentrations. For combination treatment, cells were co-treated with DDR1-IN-1 and different concentrations of cisplatin (0–32 μM) for 48 h. Combination index (CI) analysis is the most common method used to evaluate the nature of drug interactions in combination chemotherapy and provide useful quantitative information (58). CI is a numerical value calculated using the following formula: CI = C_A,X_/IC_X,A_ + C_B,X_/IC_X,B_ where C_A,X_ and C_B,X_ represent the concentrations of drugs A and B when used in combination to achieve x% drug effect. IC_X,A_ and IC_X,B_ represent the concentrations required for individual monotherapies to achieve the same x% effect. CI < 1 indicates synergy, CI = 1 indicates an additive effect, and CI > 1 represents antagonism (2).

**Plasmid construction and site-directed mutagenesis**

The DDR1 PCR product was cloned between the *Kpn*I and *Xho*I sites of the pGL4 vector. Primers with the following sequences were used: forward 5′- GGTACCCCCTGCTTCTGCCTCTTTCT-3′ and reverse 5′- CTCGAGAGGGGAGGCTGAGAACTGT -3′. The resulting constructs were confirmed using DNA sequencing. Site-directed mutagenesis was used to generate DDR1–180/+1 promoter constructs containing E2F1 mutant-binding sites using the following complementary oligonucleotides: forward 5′- ACCCCCAATGTTGCTTTCTG -3′ and reverse 5′- CAGAAAGCAACATTGGGGGT -3′. The resulting constructs were confirmed using DNA sequencing.

**Luciferase reporter assays**

Luciferase assays were conducted 48 h after transfection using a Dual-Luciferase Reporter Assay System (Promega). Normalized luciferase activity is reported as the ratio of luciferase activity to β-galactosidase activity.

**Chromatin immunoprecipitation assays**

Native protein–DNA complexes were cross-linked by treatment with 1% formaldehyde for 15 min, and chromatin immunoprecipitation (ChIP) assays were performed as previously reported (1). Briefly, equal amounts of isolated chromatin were immunoprecipitated using anti-E2F1 and IgG monoclonal antibodies. Primers with the following sequences were used for the ChIP assays: DDR1, forward 5′-CCCTGCTTCTGCCTCTTTCT-3′, and reverse, 5′-AGGGGAGGCTGAGAACTG T-3′.

**Annexin V-binding assay for apoptosis**

After the cells were exposed to cisplatin, the degree of apoptosis was measured using a FITC Annexin V Apoptosis Detection Kit, according to the manufacturer’s protocol (BD Pharmingen, Bedford, MA). The harvested cell suspension was incubated with annexin V for 15 min at room temperature in the dark and then analyzed by flow cytometry.

**Colony formation assay**

Cells (300 per well) were cultured overnight in 6-well plates in complete media. After incubation, the culture media were replaced with fresh media containing DDR1-IN-1 or cisplatin for 48 h. Treated cells were cultured in fresh media supplemented with 10% FBS for another 14 days for cell lines A2780/V and A2780/COL4A6. At the end of culturing, the cells were stained with 0.01% crystal violet for 1 h at room temperature. The figures of colony formation studies on the entire plate are shown in Supplementary Figure S2.

**References**

1. Wu YH, Chang TH, Huang YF, Huang HD, Chou CY. COL11A1 promotes tumor progression and predicts poor clinical outcome in ovarian cancer. Oncogene (2014) 33: 3432–40.
2. Chou TC, Talalay P. Quantitative analysis of dose-effect relationships: the combined effects of multiple drugs or enzyme inhibitors. Adv Enzyme Regul (1984) 22: 27–55.

**Supplementary Information**

**Supplementary Fig. S1. COL4A6 regulates cell sensitivity to cisplatin via the DDR1/NF-κB axis.** (A) Upper panel: OVCAR-8 cells were transfected with COL4A6 knockdown plasmid. After 48 h, the cells were seeded into 96-well plates and treated with various concentrations of cisplatin for 48 h; subsequently, cell sensitivity to cisplatin was measured using the MTT assay. Lower panel: OVCAR-4 cells were transfected with COL4A6-expression plasmid. After 48 h, the cells were seeded into a 96-well plate and treated with various concentrations of cisplatin for 48 h; subsequently, cell sensitivity to cisplatin was measured using the MTT assay. All experiments were performed in triplicate. *P* values were determined using the Student’s *t*-test. ^*^*P* < 0.05 or ^**^*P* < 0.005, relative to control cells treated with shControl or V. (B) OVCAR-8 cells were transfected with COL4A6 knockdown plasmid and a DDR1 cDNA plasmid, and OVCAR-4 cells were transfected with a COL4A6 cDNA plasmid and DDR1 knockdown plasmid. COL4A6,DDR1, p-DDR1, p-IKKβ, p-IKKγ, p65, COX2, and the nuclear p65 fraction in whole lysates of both types of cells were evaluated by western blotting. β-actin and SP1 were detected as loading controls for whole-cell lysates and nuclear fractions, respectively. (C) OVCAR-8 cells were transfected with different quantities of a COL4A6 knockdown plasmid (1 and 3µg) and a DDR1 cDNA plasmid (3µg), and OVCAR-4 cells were transfected with different quantities of a COL4A6 plasmid (1 and 3µg) and DDR1 knockdown plasmid (3µg). Luciferase activities were measured and normalized to Renilla luciferase activities. All experiments were performed in triplicate. *P* values were determined using the Student’s *t*-test. ^*^*P* < 0.05 or ^**^*P* < 0.005, relative to control cells treated with shControl or V.

**Supplementary Fig. S2.** Whole plates of colony formation.

**Supplementary Fig. S3. (**A) The levels of COL4A6 in vector or COL4A6 stably expressing A2780 cells were evaluated by western blotting. β-actin was used as a loading control. (B) A female 6-week-old NOD-SCID mice inoculated intraperitoneally with A2780/COL4A6 was sacrificed on day 22 after injection. (C) A female 6-week-old NOD-SCID mice inoculated intraperitoneally with A2780/COL4A6 was sacrificed on day 41 after injection. (D) Representative photo showed no visible tumor spread in the peritoneal cavity, except for a tumor in the ovaries.

**Supplementary Fig. S4.** The expression levels of COL4A6, DDR1 and p-DDR1 in A2780/V, A2780/COL4A6, and A2780 cultured with condition medium of A2780CP70 cells were evaluated using western blotting. β-Actin was used as a loading control.

**Supplementary Fig. S5.** Western blots raw data.
