# Supplementary material for: Collagen type IV alpha 6 promotes tumor progression and chemoresistance in ovarian cancer by activating the discoidin domain receptor 1 pathway
Source: Oncogenesis. 2025 Jul 2;14(1):23. doi: 10.1038/s41389-025-00565-2 (PMC12222940; doi:10.1038/s41389-025-00565-2)
Supplement: Supplementary file 2 — Supplementary figure 1 [file 41389_2025_565_MOESM2_ESM.ppt]

## Slide 1
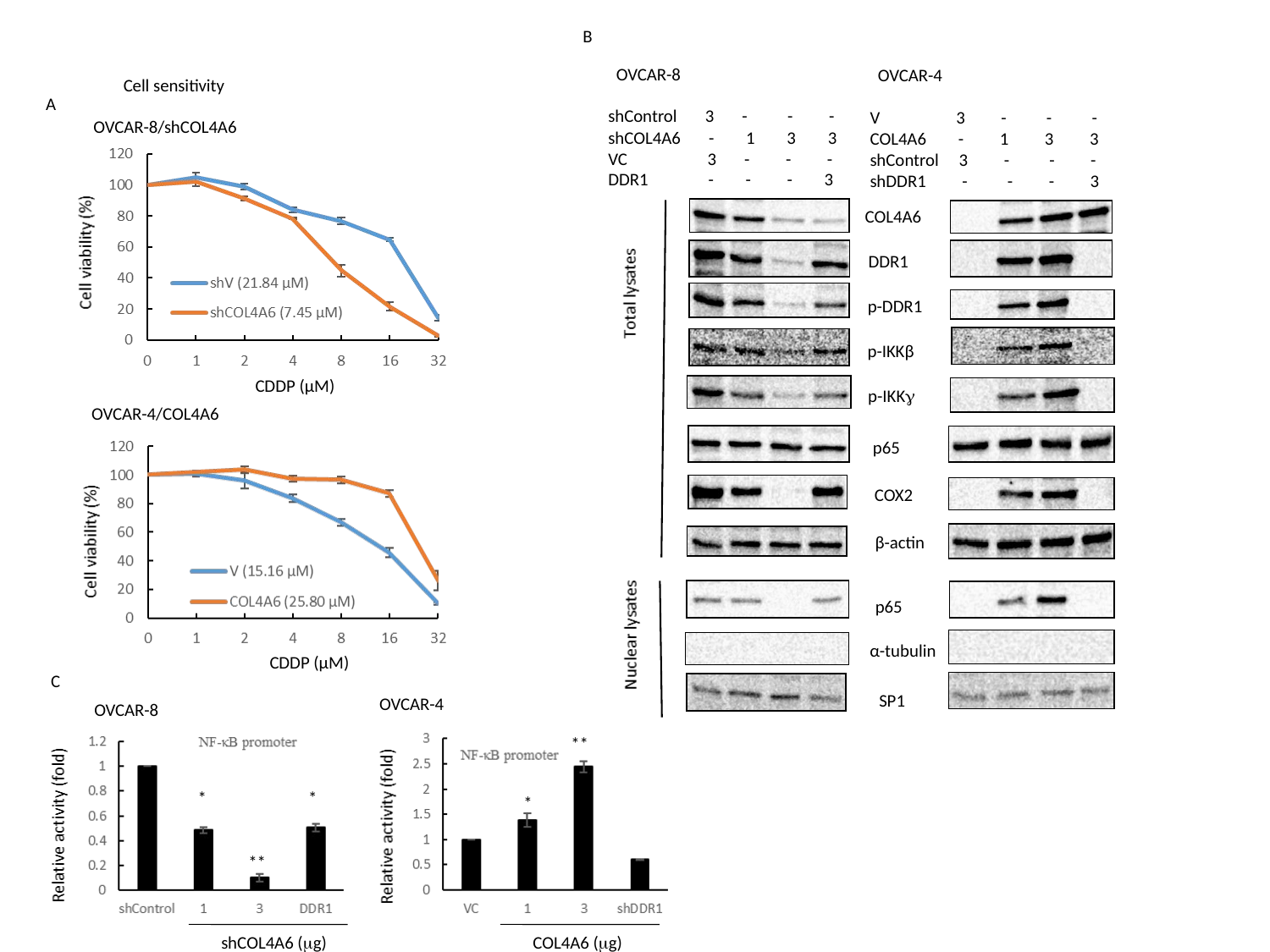

B
 OVCAR-8
shControl 3 - - -
shCOL4A6 - 1 3 3
VC 3 - - -
DDR1 - - - 3
 OVCAR-4
V 3 - - -
COL4A6 - 1 3 3
shControl 3 - - -
shDDR1 - - - 3
Cell sensitivity
A
OVCAR-8/shCOL4A6
COL4A6
DDR1
p-DDR1
p-IKKβ
CDDP (µM)
p-IKKγ
OVCAR-4/COL4A6
p65
COX2
β-actin
p65
α-tubulin
CDDP (µM)
C
SP1
OVCAR-4
OVCAR-8
**
*
*
*
**
shCOL4A6 (μg)
COL4A6 (μg)
