# Supplementary figures and images for: Collagen type IV alpha 6 promotes tumor progression and chemoresistance in ovarian cancer by activating the discoidin domain receptor 1 pathway
Source: Oncogenesis. 2025 Jul 2;14(1):23. doi: 10.1038/s41389-025-00565-2 (PMC12222940; doi:10.1038/s41389-025-00565-2)

## Slide 1
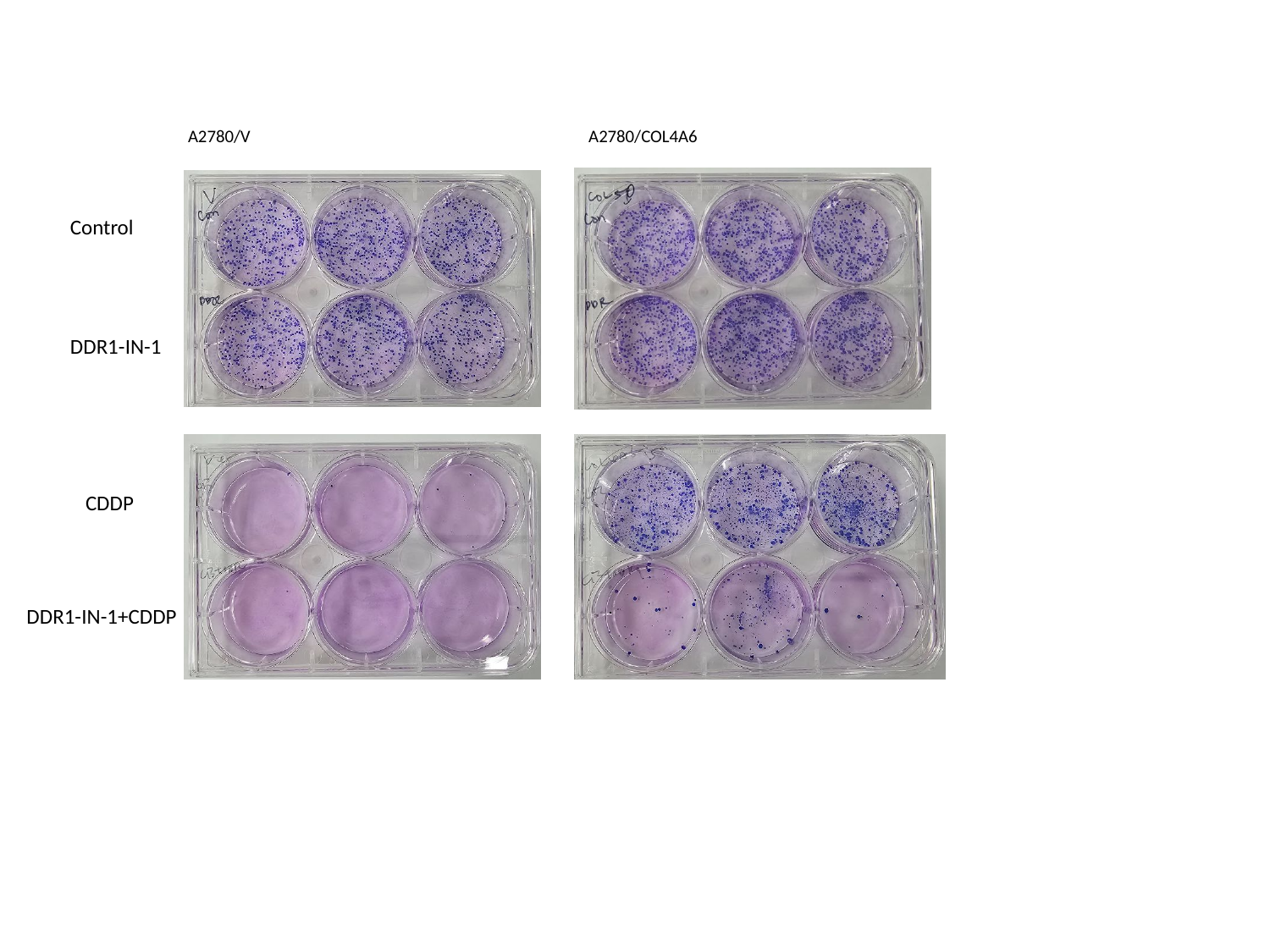

A2780/COL4A6
A2780/V
Control
DDR1-IN-1
CDDP
DDR1-IN-1+CDDP

Supplement: Supplementary file 3 — Supplementary figure 2 [file 41389_2025_565_MOESM3_ESM.ppt]

## Slide 1
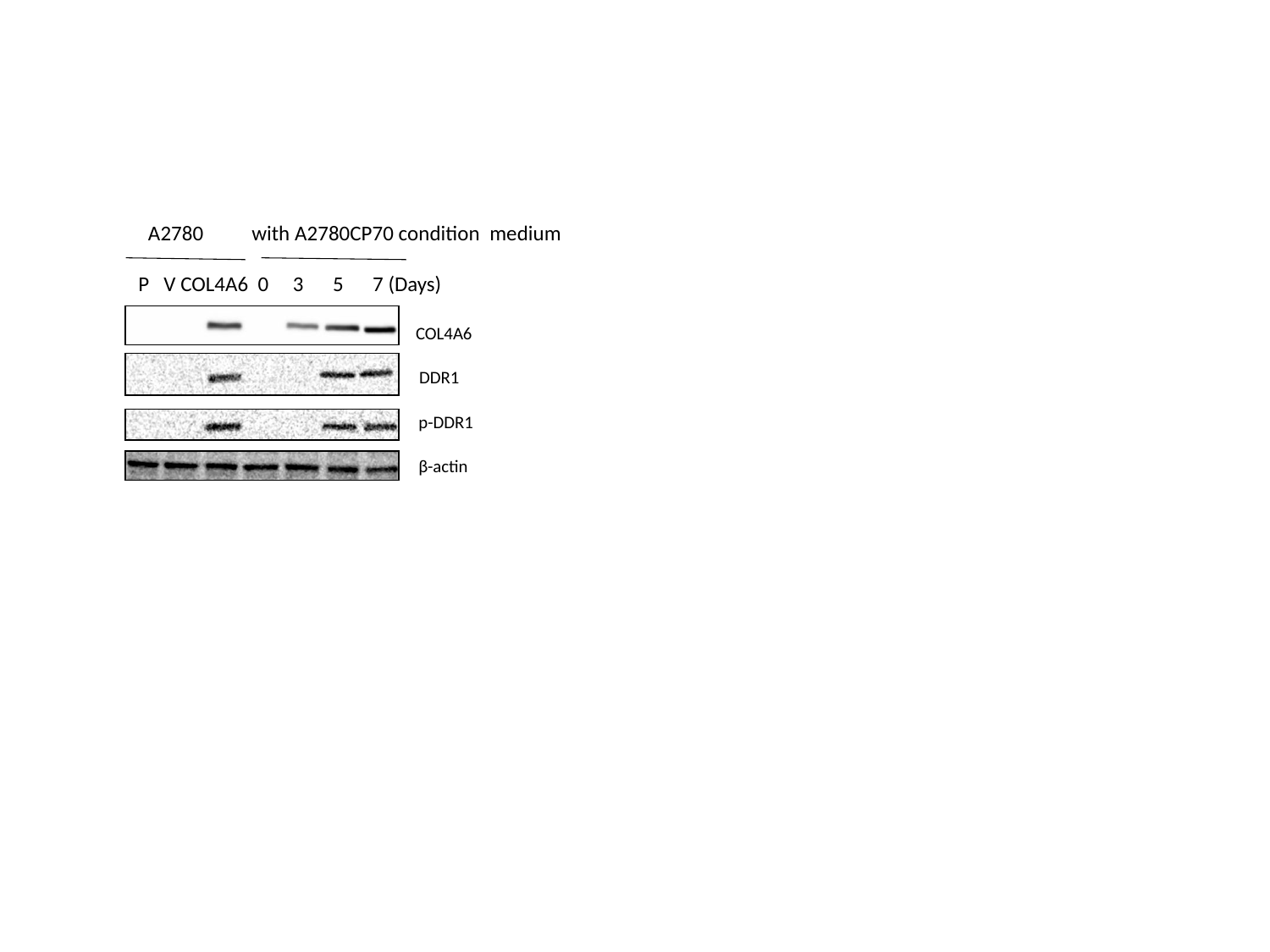

A2780 with A2780CP70 condition medium
P V COL4A6 0 3 5 7 (Days)
COL4A6
DDR1
p-DDR1
β-actin

Supplement: Supplementary file 5 — Supplementary figure 4 [file 41389_2025_565_MOESM5_ESM.ppt]
