# Supplementary material for: Collagen type IV alpha 6 promotes tumor progression and chemoresistance in ovarian cancer by activating the discoidin domain receptor 1 pathway
Source: Oncogenesis. 2025 Jul 2;14(1):23. doi: 10.1038/s41389-025-00565-2 (PMC12222940; doi:10.1038/s41389-025-00565-2)
Supplement: Supplementary file 4 — Supplementary figure 3 [file 41389_2025_565_MOESM4_ESM.pptx]

## Slide 1
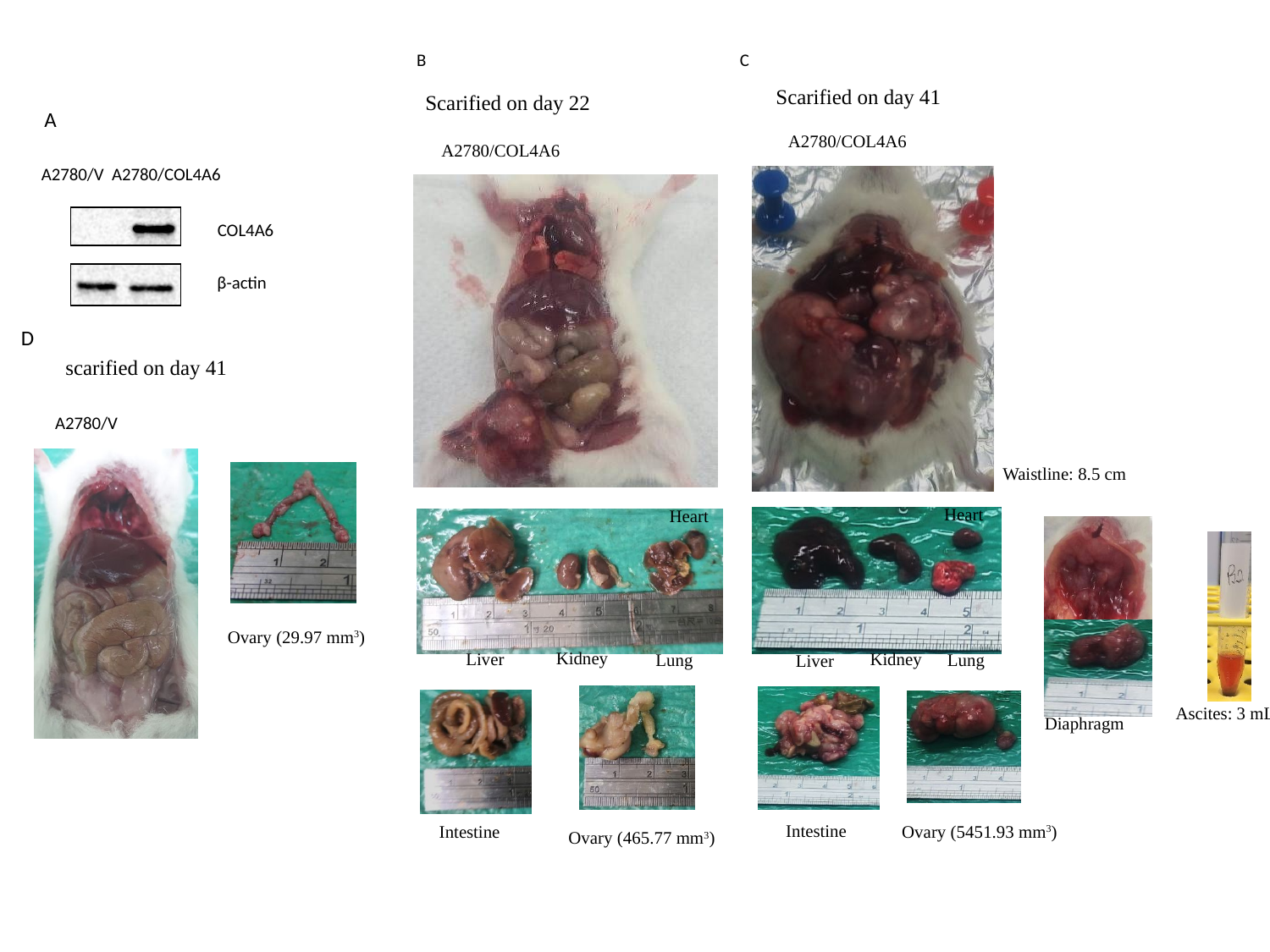

B
C
Scarified on day 41
Scarified on day 22
A
A2780/COL4A6
A2780/COL4A6
A2780/V A2780/COL4A6
COL4A6
β-actin
D
scarified on day 41
A2780/V
Waistline: 8.5 cm
Heart
Heart
Ovary (29.97 mm3)
Kidney
Liver
Kidney
Lung
Lung
Liver
Ascites: 3 mL
Diaphragm
Intestine
Intestine
Ovary (5451.93 mm3)
Ovary (465.77 mm3)
