# Supplementary material for: Collagen type IV alpha 6 promotes tumor progression and chemoresistance in ovarian cancer by activating the discoidin domain receptor 1 pathway
Source: Oncogenesis. 2025 Jul 2;14(1):23. doi: 10.1038/s41389-025-00565-2 (PMC12222940; doi:10.1038/s41389-025-00565-2)
Supplement: Supplementary file 6 — Supplementary figure 5 [file 41389_2025_565_MOESM6_ESM.pptx]

## Slide 1
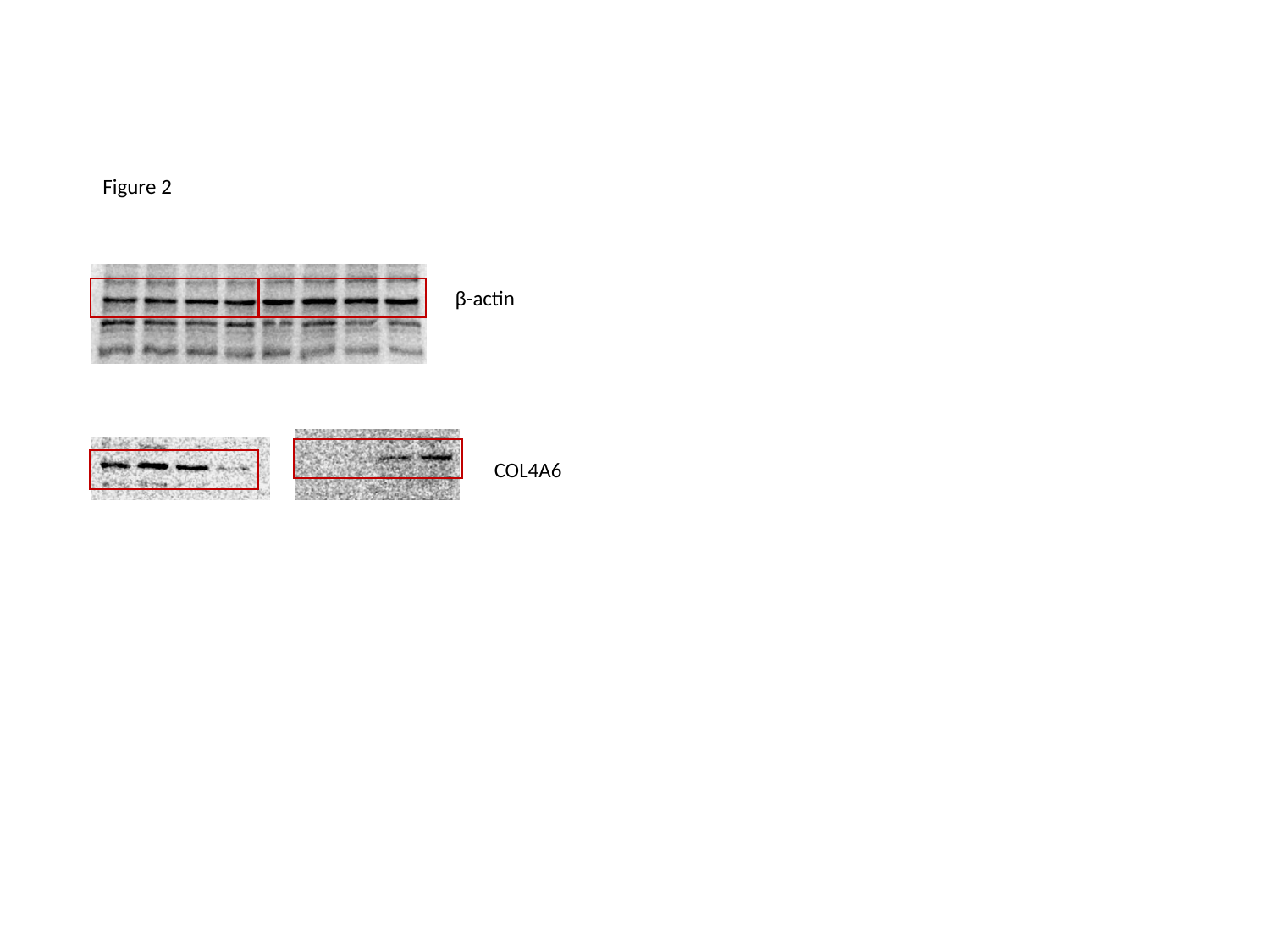

Figure 2
β-actin
COL4A6

## Slide 2
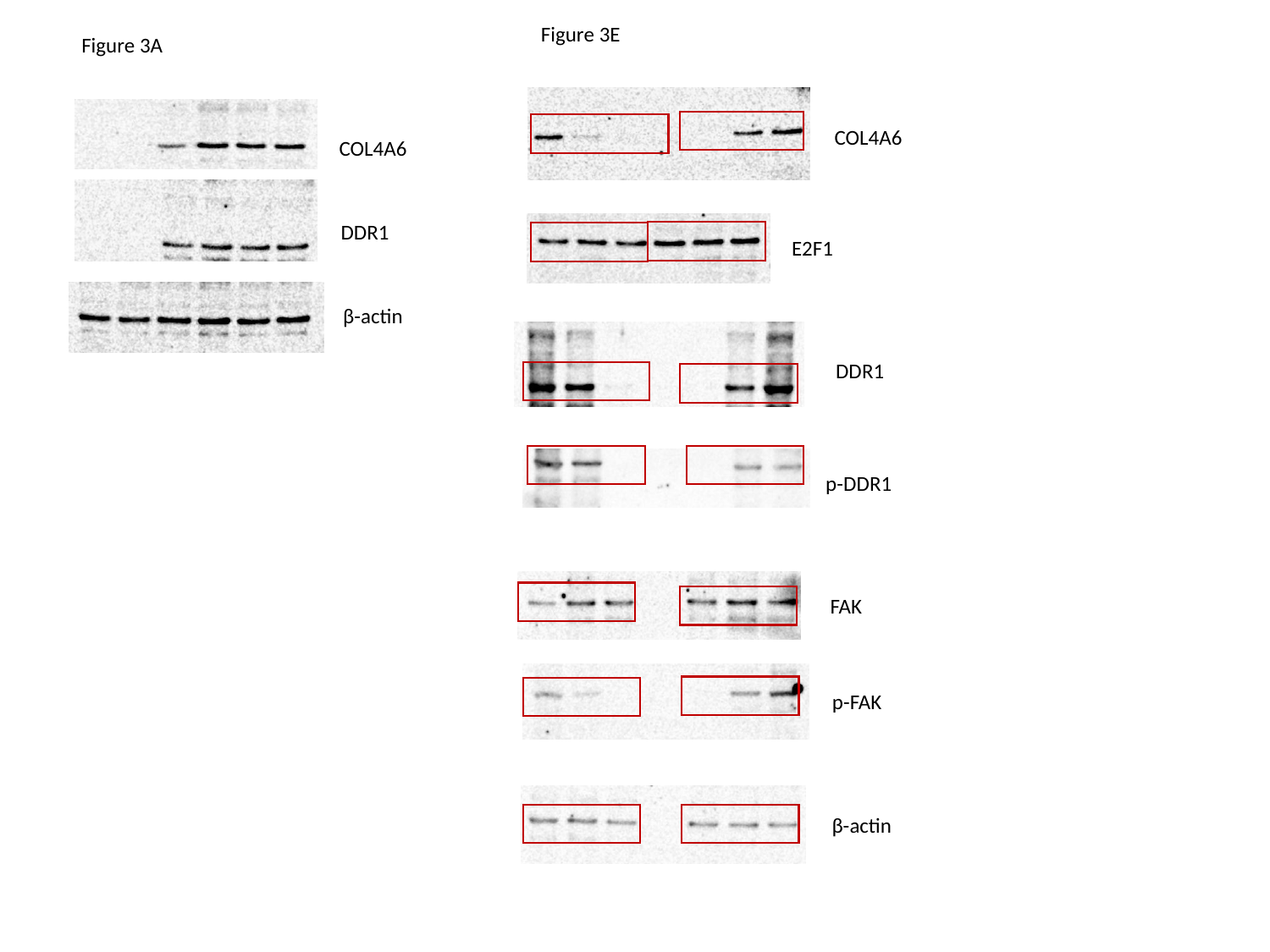

Figure 3E
Figure 3A
COL4A6
COL4A6
DDR1
E2F1
β-actin
DDR1
p-DDR1
FAK
p-FAK
β-actin

## Slide 3
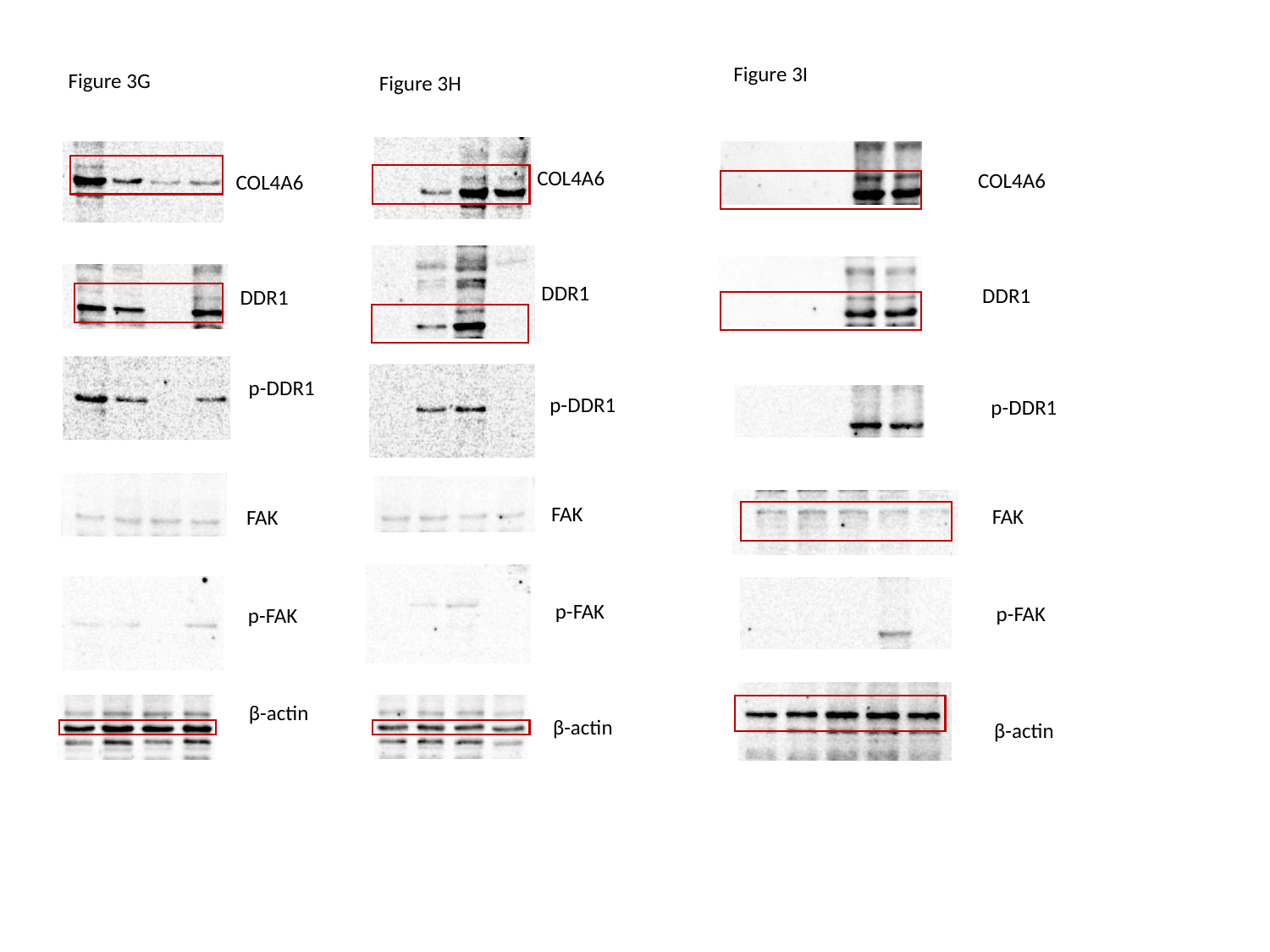

Figure 3I
Figure 3G
Figure 3H
COL4A6
COL4A6
COL4A6
DDR1
DDR1
DDR1
p-DDR1
p-DDR1
p-DDR1
FAK
FAK
FAK
p-FAK
p-FAK
p-FAK
β-actin
β-actin
β-actin

## Slide 4
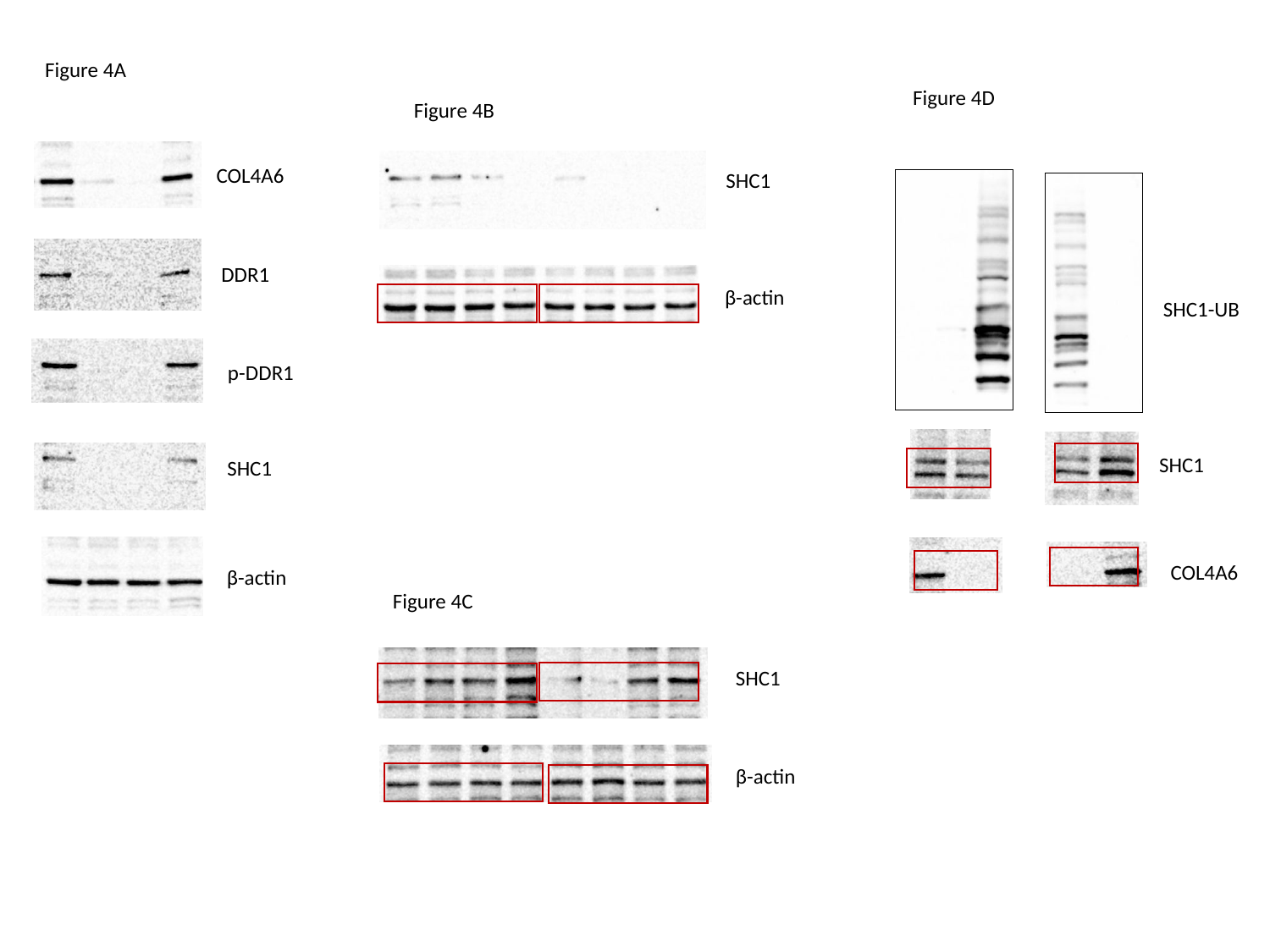

Figure 4A
Figure 4D
Figure 4B
COL4A6
SHC1
DDR1
β-actin
SHC1-UB
p-DDR1
SHC1
SHC1
COL4A6
β-actin
Figure 4C
SHC1
β-actin

## Slide 5
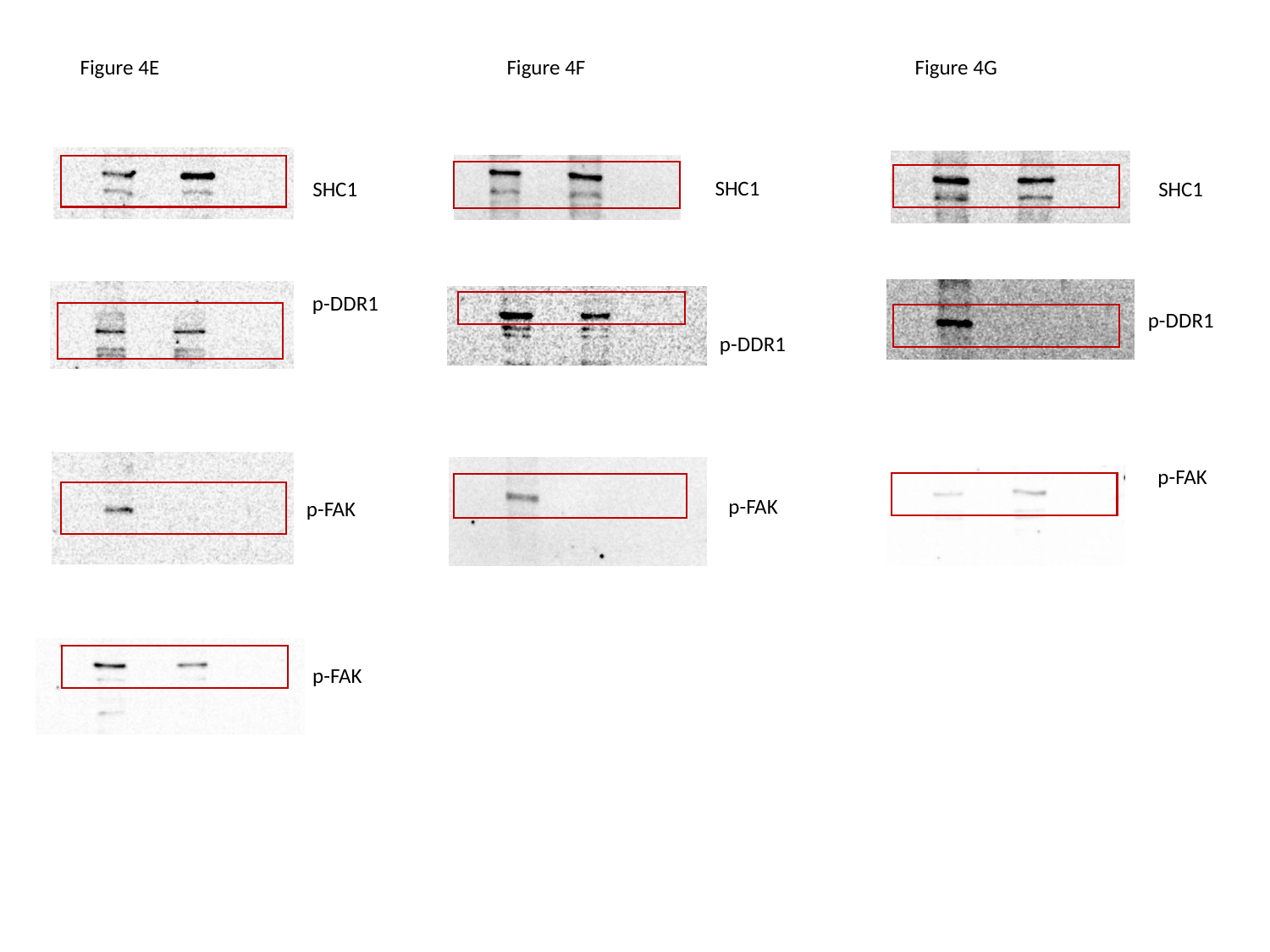

Figure 4G
Figure 4E
Figure 4F
SHC1
SHC1
SHC1
p-DDR1
p-DDR1
p-DDR1
p-FAK
p-FAK
p-FAK
p-FAK

## Slide 6
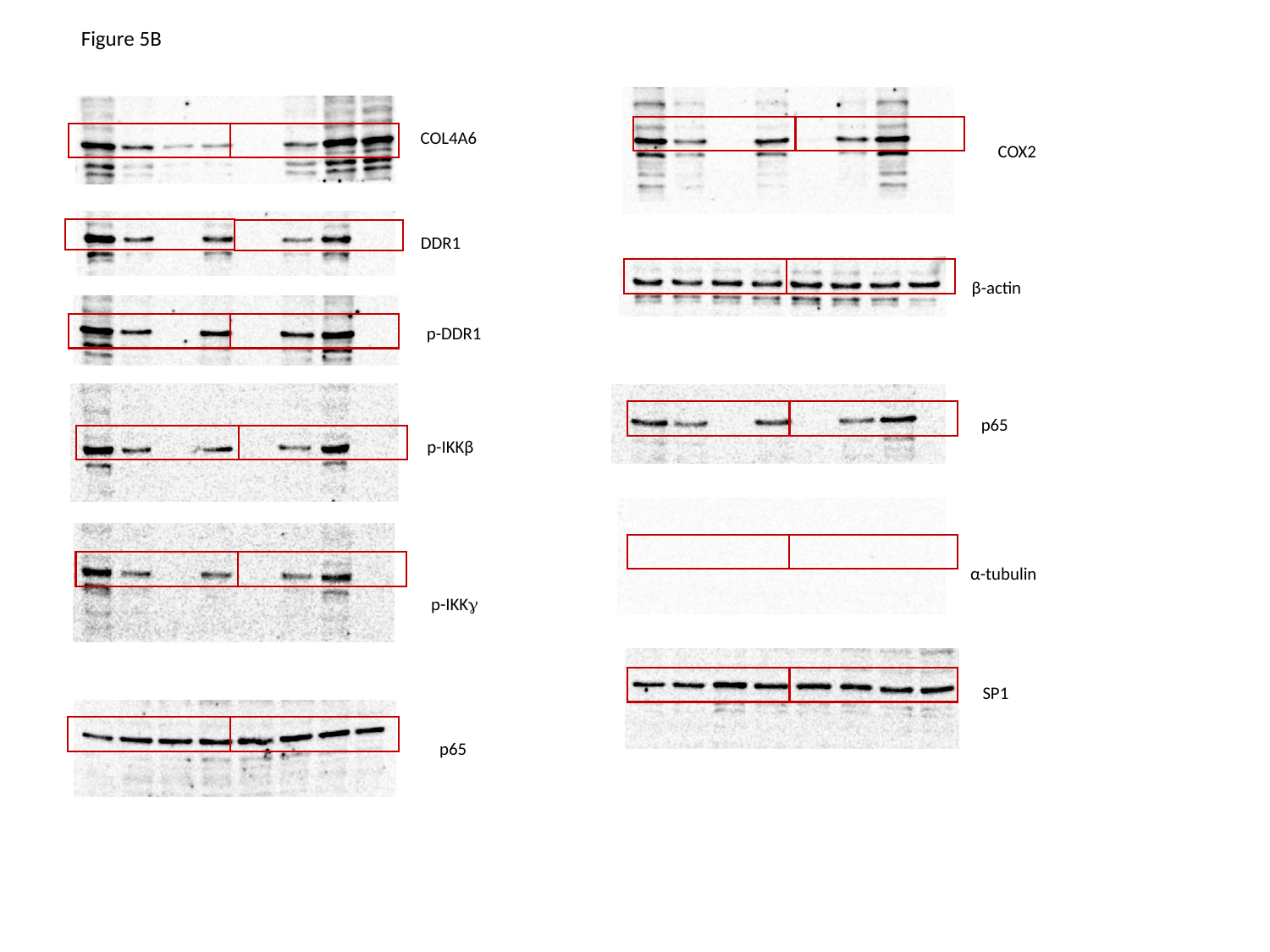

Figure 5B
COL4A6
COX2
DDR1
β-actin
p-DDR1
p65
p-IKKβ
α-tubulin
p-IKKγ
SP1
p65

## Slide 7
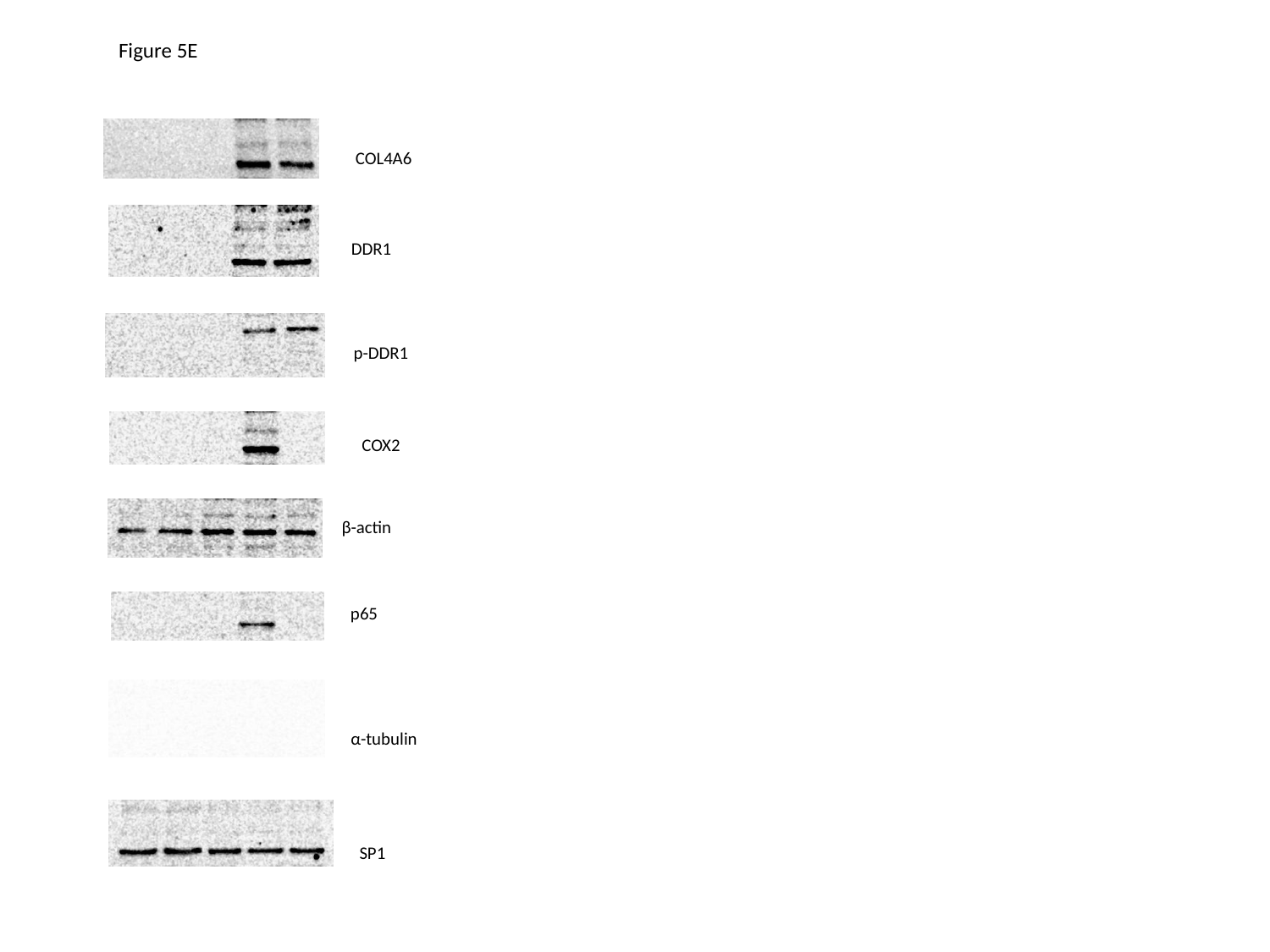

Figure 5E
COL4A6
DDR1
p-DDR1
COX2
β-actin
p65
α-tubulin
SP1

## Slide 8
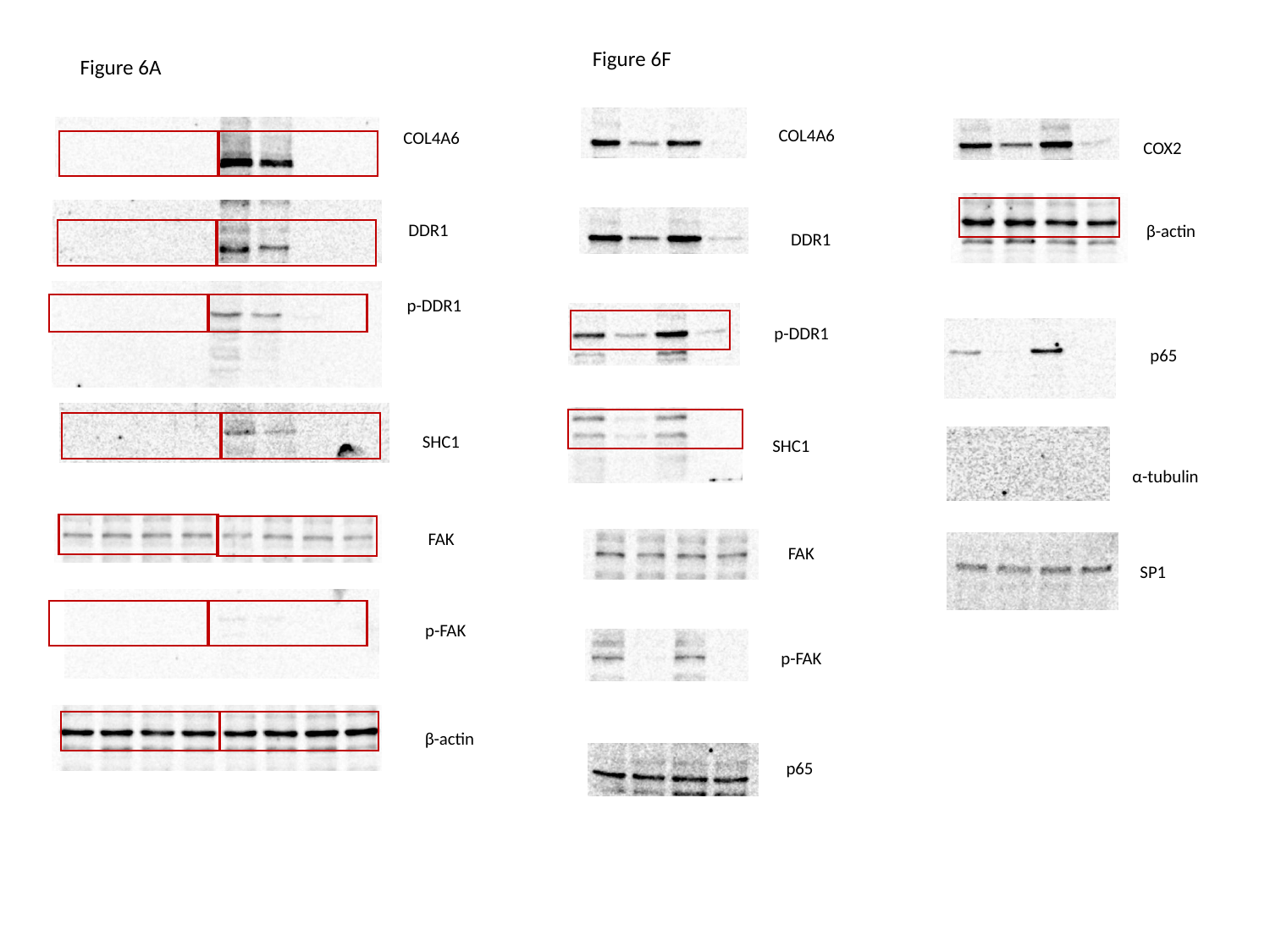

Figure 6F
Figure 6A
COL4A6
COL4A6
COX2
DDR1
β-actin
DDR1
p-DDR1
p-DDR1
p65
SHC1
SHC1
α-tubulin
FAK
FAK
SP1
p-FAK
p-FAK
β-actin
p65

## Slide 9
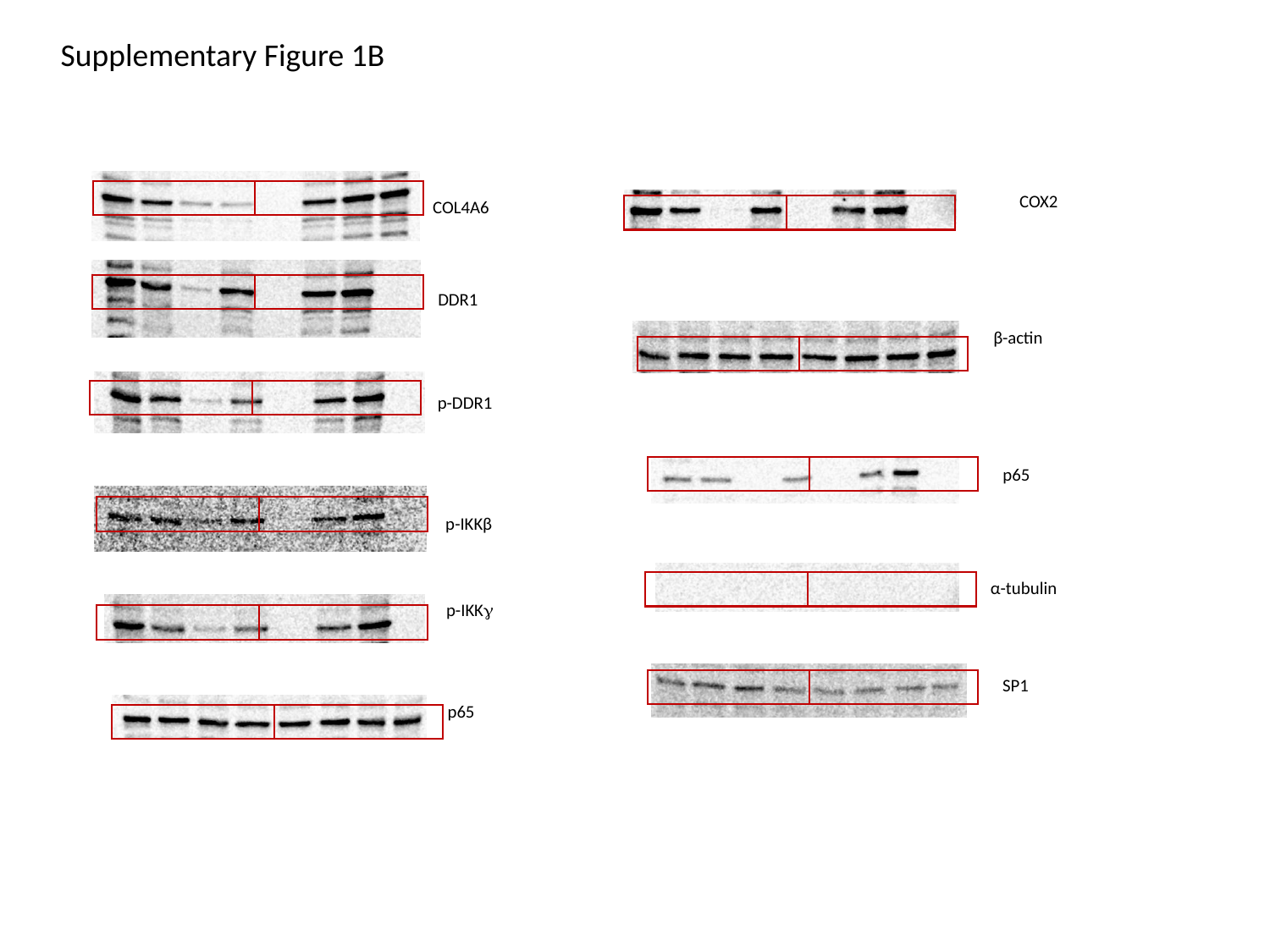

Supplementary Figure 1B
COX2
COL4A6
DDR1
β-actin
p-DDR1
p65
p-IKKβ
α-tubulin
p-IKKγ
SP1
p65

## Slide 10
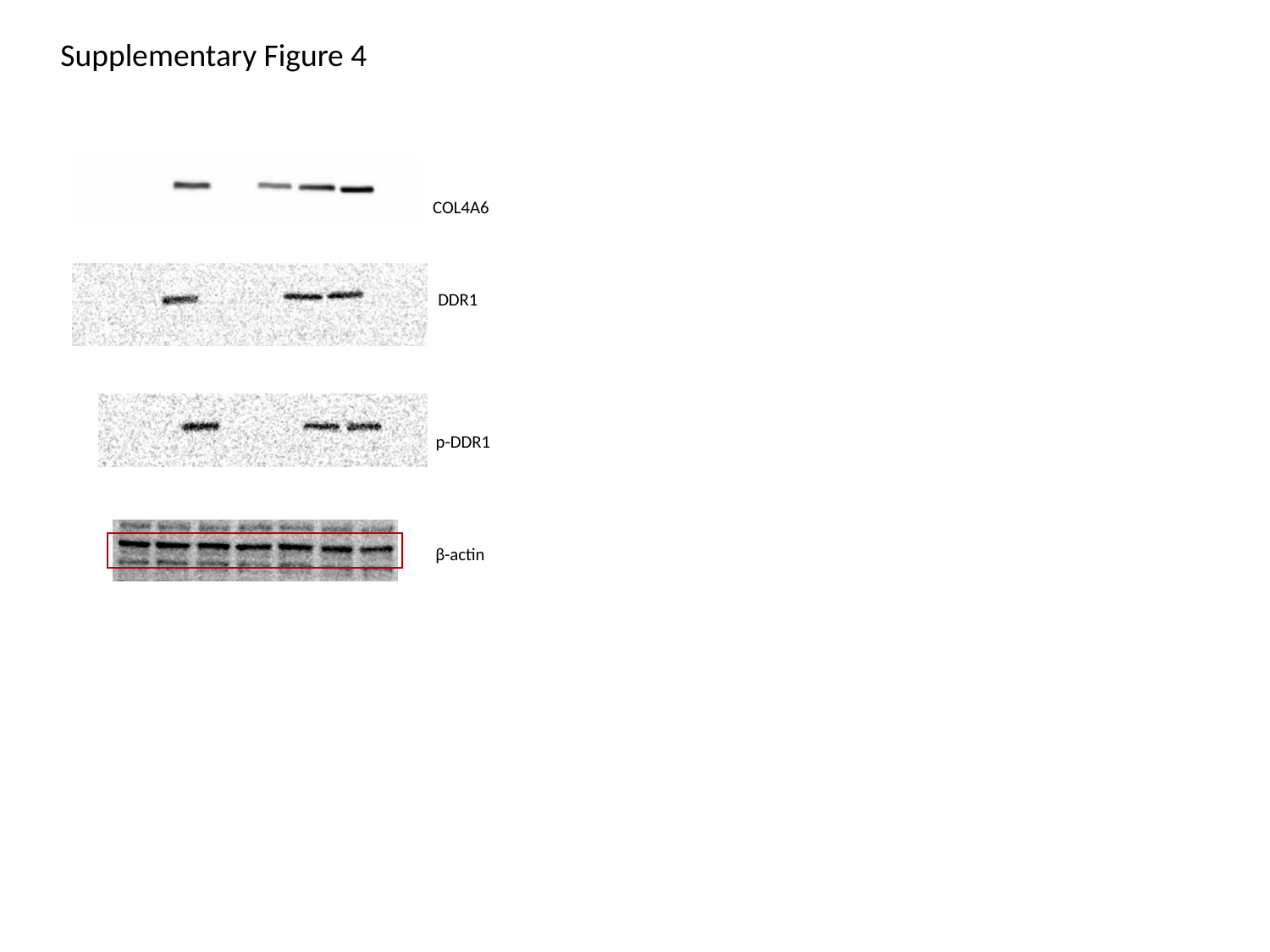

Supplementary Figure 4
COL4A6
DDR1
p-DDR1
β-actin
